# A Pancreatitis‐Inspired Trypsinogen Nanoplatform Reprograms Tumor‐Associated Macrophages via NF‐κB for Pancreatic Cancer Immunotherapy

**DOI:** 10.1002/advs.76386

**Published:** 2026-06-27

**Authors:** Lei Cao, Yu He, Wenhao Li, Bo Gong, Han Lin, Jianlin Shi, Yuan Yong, Wencheng Wu

**Affiliations:** ^1^ Department of Radiology Sichuan Academy of Medical Sciences Sichuan Provincial People's Hospital University of Electronic Science and Technology of China Chengdu Sichuan P. R. China; ^2^ Nanomedicine Innovation Research and Transformation Institute Affiliated Hospital of North Sichuan Medical College Nanchong Sichuan P. R. China; ^3^ Central Laboratory and Department of Medical Ultrasound, Sichuan Academy of Medical Sciences Sichuan Provincial People’s Hospital, University of Electronic Science and Technology of China Chengdu Sichuan P. R. China; ^4^ Shanghai Institute of Ceramics, Chinese Academy of Sciences Shanghai P. R. China

**Keywords:** cancer immunotherapy, immune reprogramming, macrophage polarization, pancreatic cancer

## Abstract

Although reprogramming tumor‐associated macrophages (TAMs) represents a promising therapeutic strategy, approaches that are both precise and safe remain scarce. Inspired by the pro‐inflammatory M1‐like response triggered by trypsinogen in pancreatitis, trypsinogen as a novel macrophage reprogrammer is identified. Here, it is shown that trypsinogen drives M2‐to‐M1 repolarization via NF‐κB activation. To harness this activity, a pancreatitis‐inspired nanoplatform (JT@NPs‐aCD11b) co‐delivering trypsinogen and the CD47 inhibitor JQ1 to TAMs is developed. In pancreatic cancer models, JT@NPs‐aCD11b reprogrammed TAMs to an M1 phenotype while JQ1 blocked the “do not eat me” signal on cancer cells, synergistically enhancing phagocytosis. This strategy remodeled the immunosuppressive microenvironment, increased effector immune cells, and reduced suppressive populations, leading to potent tumor suppression, prolonged survival, and durable immune memory. Macrophage depletion abrogated efficacy, confirming TAMs as the primary mediators. This work establishes trypsinogen as a versatile immunomodulator and its nanoplatform as a promising strategy for pancreatic cancer immunotherapy.

## Graphical abstract

Trypsinogen‐loaded nanoparticles reprogram TAMs via NF‐κB/NLRP3, driving M2→M1 conversion and potent phagocytosis to unleash antitumor immunity.

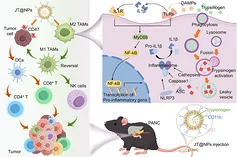

## 1 Introduction

Pancreatic ductal adenocarcinoma (PDAC) remains one of the most lethal malignancies, with a five‐year survival rate below 10% and limited response to current immunotherapies [1, 2, 3]. Central to this therapeutic resistance is the profoundly immunosuppressive tumor microenvironment (TME), characterized by a dense desmoplastic stroma and a predominance of M2‐polarized tumor‐associated macrophages (TAMs) [4, 5, 6]. These M2‐like TAMs actively suppress antitumor immunity by secreting immunosuppressive cytokines (e.g., IL‐10, TGF‐β), recruiting regulatory T cells (Tregs), and inhibiting cytotoxic CD8^+^ T cells [7, 8, 9]. In contrast, M1‐polarized macrophages exert pro‐inflammatory and antitumor functions, including antigen presentation and direct tumor cell killing [10, 11, 12]. Thus, reprogramming TAMs from an M2 to an M1 phenotype represents a rational and promising strategy to reverse immunosuppression and potentiate antitumor immunity in PDAC [13, 14, 15, 16].

The discovery of trypsinogen (Try) as a potential macrophage reprogrammer emerged from an unexpected observation in pancreatitis research [17]. During acute pancreatitis, trypsinogen is aberrantly activated within acinar cells, triggering local inflammation. However, studies have also shown that when trypsinogen is internalized by phagocytic macrophages, it elicits a robust pro‐inflammatory response characterized by elevated levels of IL‐1β, IL‐6, and TNF‐α—a cytokine signature reminiscent of M1‐polarized macrophages [18, 19, 20]. This intriguing paradox led us to hypothesize that trypsinogen might itself serve as a direct inducer of M1 macrophage polarization. Subsequent mechanistic exploration revealed that trypsinogen activates the canonical NF‐κB signaling pathway by promoting phosphorylation of the p65 subunit at serine 536, driving its nuclear translocation and the transcription of pro‐inflammatory genes [19, 20, 21, 22]. This defined molecular mechanism positions trypsinogen as a novel and specific TAM‐reprogramming agent with distinct advantages over conventional polarization inducers.

Despite its immunomodulatory potential, the systemic application of trypsinogen is hindered by several obstacles, including rapid clearance, lack of tumor specificity, and potential off‐target toxicity [23, 24, 25, 26, 27]. To overcome these limitations, we developed a targeted ionizable liposomal nanoparticle (LNP) platform (JT@NPs‐aCD11b) for the co‐delivery of trypsinogen and JQ1, a small‐molecule inhibitor of the CD47‐SIRPα “don't eat me” immune checkpoint. The nanoparticle surface was functionalized with CD11b antibodies to enable selective targeting of TAMs within the TME. Upon internalization, trypsinogen activates NF‐κB signaling to repolarize M2 TAMs toward an M1 phenotype, while JQ1 blocks CD47‐mediated immune evasion, synergistically enhancing macrophage‐mediated phagocytosis and antitumor immunity. In this study, we demonstrate that this pancreatitis‐inspired trypsinogen nanoplatform effectively reprograms TAMs via NF‐κB activation, remodels the immunosuppressive TME, and elicits robust antitumor immune responses in both subcutaneous and orthotopic pancreatic cancer models (Figure 1). This work establishes trypsinogen as a novel and potent macrophage‐polarizing agent and highlights the potential of targeted co‐delivery strategies for combinatorial immunotherapy in recalcitrant malignancies.

**FIGURE 1:**
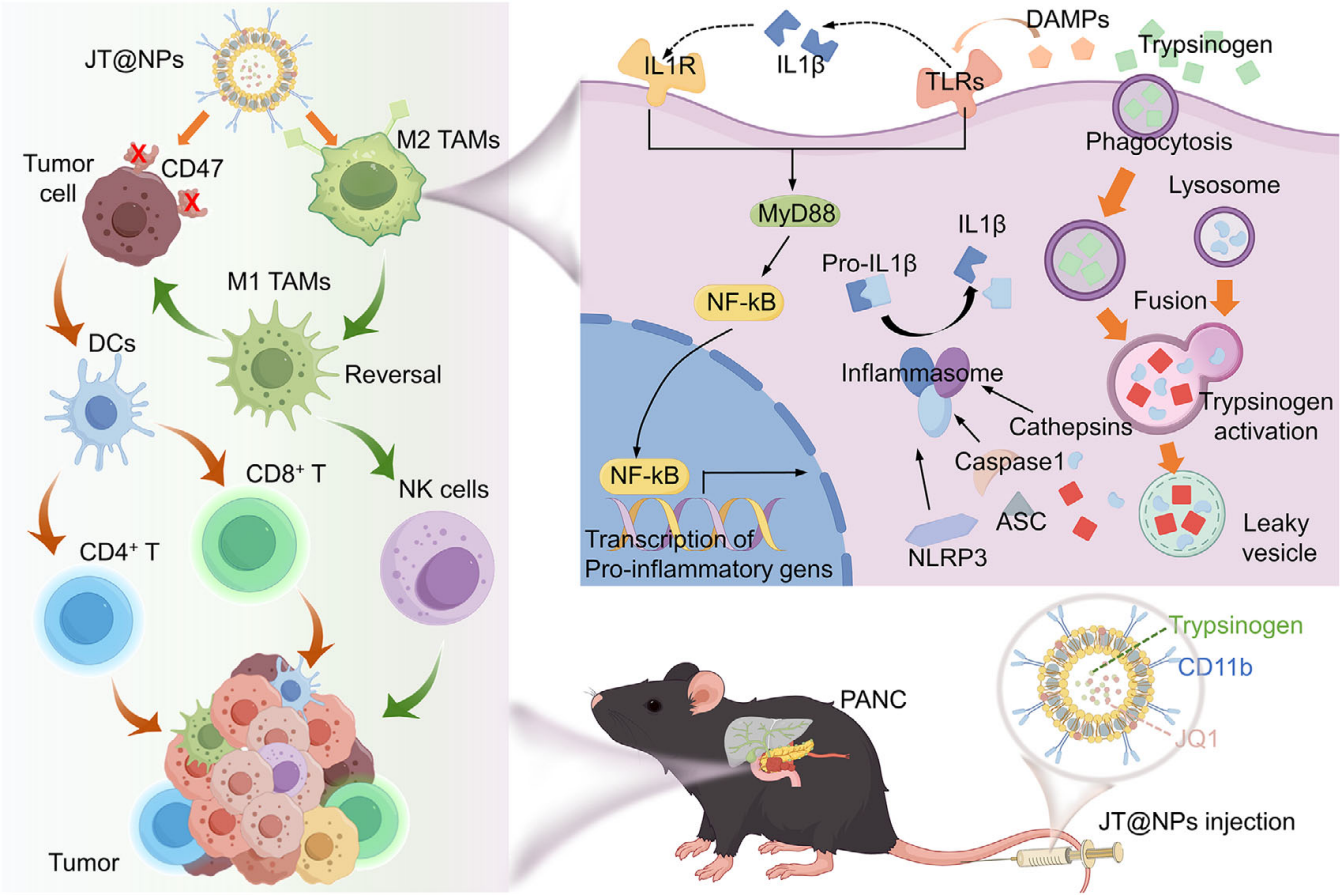
Schematic illustration of macrophage reprogramming and tumor immune microenvironment modulation mediated by JT@NPs, a smart nanoresponder co‐encapsulating Try and the CD47 inhibitor JQ‐1, for potentiated anti‐tumor immunotherapy.

## 2 Results

### 2.1 Polarization of M2 Macrophages to M1 by Try

In this study, the ability of Try (Figure 2a) to modulate macrophage polarization was investigated using bone marrow‐derived macrophages (BMDMs) polarized toward the M2 phenotype with IL‐4 or toward the M1 phenotype using interferon‐γ (IFN‐γ) and lipopolysaccharide (LPS). These polarized macrophages closely mirror the functional and phenotypic characteristics of M2‐ or M1‐like tumor‐associated macrophages (TAMs) and are thus widely used as reliable in vitro models for TAM‐related research. RNA sequencing analysis revealed that treatment of M2‐like macrophages with Try led to a marked downregulation of M2‐associated genes—including *Arg1*, *Cd163*, *Cd206*, *Fizz*, *Il‐10*, *Mmp2*, and *Smad3*—while simultaneously upregulating multiple M1‐associated genes such as *Ccl5*, *Cxcl9*, *Cxcl10*, *Nos*, *Il‐1β*, *Il‐12*, *Tlr2*, *Tlr9*, and *Tnf‐α* (Figure 2b). These transcriptional changes were further validated at the gene expression level using quantitative reverse transcription polymerase chain reaction (RT‐qPCR) and at the protein secretion level via enzyme‐linked immunosorbent assay (ELISA) (Figure 2c–j). Specifically, in Try‐treated M2 macrophages, both the expression of M2 functional markers (Arg‐1 and Fizz) and the secretion of M2‐associated cytokines (CCL22 and IL‐10) were significantly suppressed (Figure 2c,d,g,h). In contrast, the expression of M1 markers (tumor necrosis factor‐α [TNF‐α], iNOS, and IL‐12) and the release of M1‐related cytokines (TNF‐α and IL‐1β) were notably enhanced (Figure 2e,f,i,j). Heat‑inactivated trypsinogen failed to induce M2‑to‑M1 repolarization or alter M1/M2 marker expression in BMDMs (Figure S1). Flow cytometric analysis further corroborated these findings, showing a significant increase in the CD206^−^CD86^+^ cell population—representing the M1 phenotype—following Try exposure (Figure 2k,l). Additionally, morphological examination indicated that M1 and M2 macrophages exhibited distinct cellular architectures, consistent with previously reported phenotypes. Notably, Try treatment induced a structural transition in M2 macrophages, causing them to adopt a morphology resembling that of M1 macrophages (Figure 2m). Collectively, the results presented in Figure 2 demonstrate that Try effectively promotes the repolarization of M2 macrophages toward an M1‐like phenotype.

**FIGURE 2:**
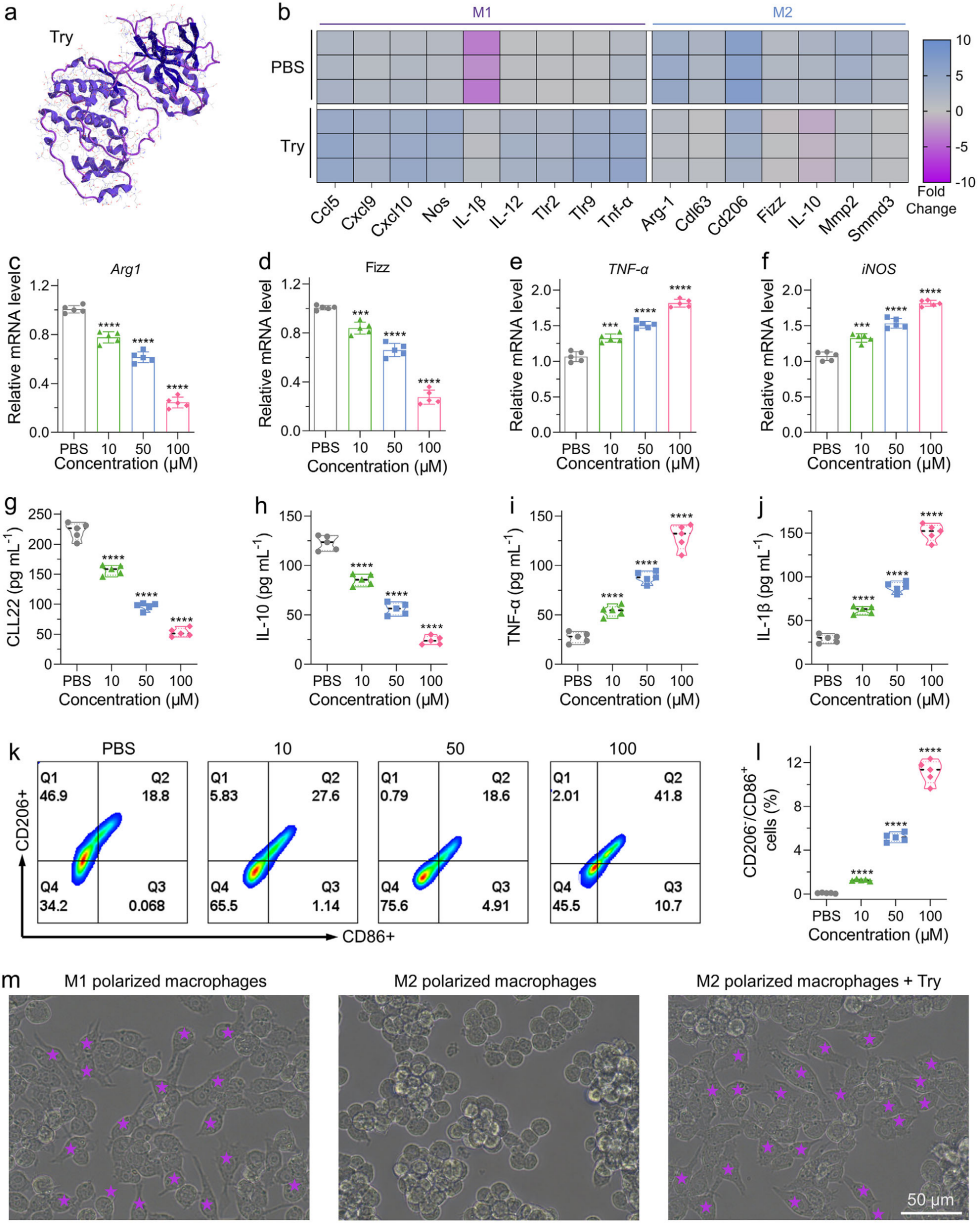
Try regulates the polarization of M2 macrophages to M1 ones. (a) Chemical structure of Try. (b) Hierarchical clustering heatmap displaying the expression patterns of M1‐ and M2‐related genes in IL‐4–stimulated bone marrow–derived macrophages (BMDMs, M2‐like phenotype) treated with phosphate‐buffered saline (PBS) or 50 mM Try. (c–j) mRNA levels of polarization markers in Try‐treated M2‐like macrophages were quantified by reverse transcription quantitative PCR (RT‐qPCR) (*n* = 5; *p < 0.05, **p < 0.01, and ****p < 0.0001 versus PBS). (k,l) Flow cytometric analysis of macrophage phenotypes and corresponding quantitation following PBS or Try treatment, gated on F4/80^+^CD86^+^ cells for M1 and F4/80^+^CD206^+^ cells for M2 polarization (*n* = 5; ***p < 0.001 and ****p < 0.0001 compared to PBS). (m) Representative morphological images of BMDMs under different polarization conditions: M1 macrophages induced by IFN‐γ and LPS co‐stimulation, IL‐4–induced M2 macrophages, and M2 macrophages treated with 50 mM Try.

### 2.2 Macrophage Polarization by Try via Activation of NF‐κB Pathway

The mechanisms underlying the efficacy of Try in macrophage polarization were subsequently investigated using M2 macrophages. First, we performed gene‐level differential expression analysis on Try‐treated M2 macrophages using bulk RNA sequencing (RNA‐seq). Volcano plot analysis revealed that more than 1500 differentially expressed genes were identified in trypsin‐treated M2 macrophages compared to untreated macrophages, with 838 genes significantly upregulated (Figure 3a). Gene Ontology (GO) classification of these upregulated genes (Figure 3b and Figure S2a) indicated significant enrichment in immune response, cytokine production, and innate immune response‐related cytological events in Try‐treated M2 macrophages. Furthermore, KEGG pathway enrichment analysis revealed that the nuclear factor‐κB (NF‐κB) signaling pathway was significantly upregulated in Try‐stimulated M2 macrophages, along with a subsequent significant upregulation of the downstream TNF‐α pathway (Figure 3c and Figure S2b). These results indicate that Try‐induced alterations in the M2 macrophage phenotype are most likely mediated through the activation of inflammation‐related pathways, particularly the NF‐κB pathway. We then performed a detailed analysis of the NF‐κB molecular pathway using immunofluorescence techniques. As shown in Figure 3d, Cy5.5‐labeled Try was rapidly internalized by M2 macrophages. Following internalization, trypsinogen was activated by cathepsin B (CTSB) in M2 macrophages. The resulting active trypsin destabilized the cathepsin‐containing phagolysosomes, promoting the activation of the NLRP3 inflammasome and caspase‐1, as well as the release of IL‐1β (Figure 3e–g and Figure S3). Autocrinally, the secreted IL‐1β engaged its receptor on macrophages, driving NF‐κB nuclear translocation and reinforcing a pro‐inflammatory circuit. Robust nuclear translocation of P‐NF‐κB p65 was observed in Try‐treated M2 macrophages versus untreated controls, as evidenced by immunofluorescence imaging and confirmed by single‐cell fluorescence linear analysis (Figure 3h,i). Western blot (WB) analysis further confirmed NF‐κB pathway activation (Figure 3j and Figure S4), consequently polarizing macrophage markers from an M2 to an M1 phenotype and leading to a significant increase in TNF‐α and IFN‐γ levels (Figure 3k). To further verify the proposed mechanism, we performed a series of inhibitor experiments in M2‐polarized bone marrow‐derived macrophages (BMDMs). Prior to Try stimulation, cells were pretreated with CA‐074 Me (cathepsin B inhibitor, 50 µM), MCC950 (NLRP3 inflammasome inhibitor, 50 µM), IL‐1 receptor antagonist (IL‐1Ra, 25 nM), or BAY 11‐7082 (NF‐κB inhibitor, 10 µM). ELISA results showed that CA‐074 Me significantly suppressed Try‐induced IL‐1β and TNF‐α secretion, confirming cathepsin B‐mediated Try activation as an essential initiating event (Figure S5a,b). MCC950 markedly attenuated IL‐1β release and partially reduced TNF‐α production, indicating a critical role of NLRP3 inflammasome activation (Figure S5c,d). IL‐1Ra abrogated the Try‐driven production of TNF‐α and IFN‐γ, establishing that IL‐1β acts in an autocrine manner to amplify macrophage activation (Figure S5e,f). BAY 11‐7082 nearly abolished both TNF‐α and IFN‐γ, confirming that NF‐κB is the central downstream transcriptional mediator of this pathway (Figure S5g,h). Thus, intra‐macrophage Try activation can result in an autocrine loop in macrophage activation that further enhances the pro‐inflammatory response (Figure 3l). This process provides a basis for Try to remodel tumor‐associated M2 macrophages, exerting antitumor effects.

**FIGURE 3:**
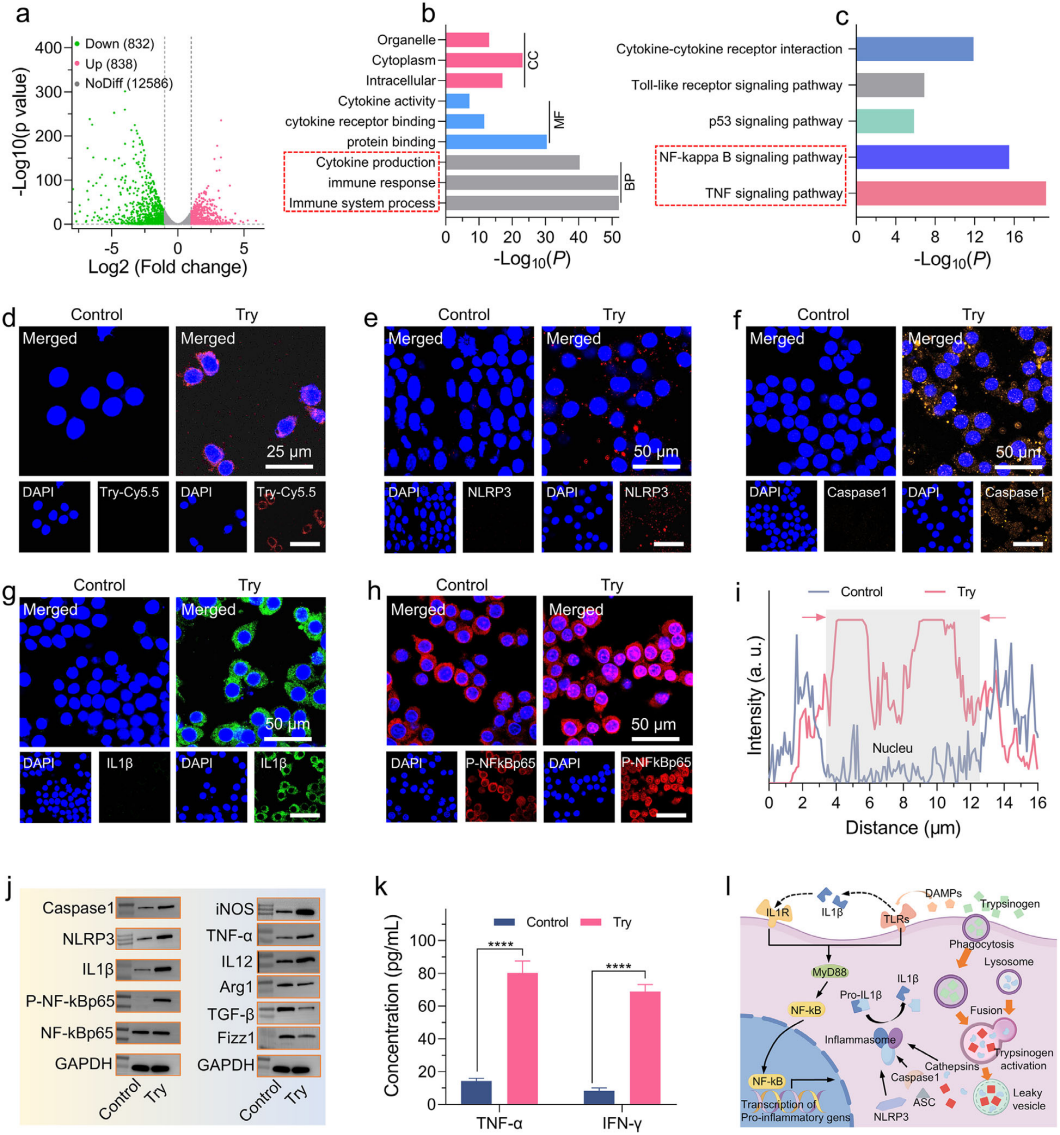
Try induces the polarization of M2 macrophages to M1 ones by activating the NF‐κB pathway. (a) Volcano plot indicating over 1670 differentially expressed genes between Try‐ and PBS‐treated macrophages. Threshold P_adj_  =  0.01. P_adj_, adjusted *p* value. (b, c) Significantly overexpressed cytological events and pathways in Try‐macrophages in comparison with untreated macrophages. (d) Confocal laser scanning microscopy image of macrophages after co‐incubation with PBS and Cy5.5‐labeled Try. The activity of the NF‐κB signaling pathway in M2 macrophages under different treatment conditions was analyzed using immunofluorescence technology for (e) NLRP3, (f) Caspase1, (g) IL‐1β, and (h) phosphorylated NF‐κB at position 65 (P‐NFκB65). (i) Linear distribution analysis of P‐NFκBp65 fluorescence signals in Try‐ and PBS‐treated macrophages. (j) The expression of NF‐κB and different biomarkers in Try‐ and PBS‐treated treated macrophages was analyzed using western blot assay (WB). The quantification was shown in Figure S3. (k) The expression of proinflammatory cytokines (TNF‐α and IFN‐γ) in macrophages was measured using enzyme‐linked immunosorbent assay (ELISA) (*n* = 3; ****p< 0.0001). (l) The schematic of signaling pathways associated with Try‐induced macrophage polarization from M2 to M1.

### 2.3 Fabrication and Physicochemical Characterization of Nanoformulation

In this study, a macrophage‐targeted, anti‐CD11b antibody‐coated, ionizable lipid nanoparticle (LNP) was developed using microfluidic technology (Figure 4a) to facilitate the in vivo co‐delivery of Try and the CD47 inhibitor JQ1 within the tumor microenvironment (TME). Following the surface coating of JQ1/Try co‐loaded LNPs (termed JT@NPs) with the anti‐CD11b antibody, the targeted nanoformulation (termed JT@NPs‐aCD11b) demonstrated a uniform spherical morphology, as observed in transmission electron microscopy (TEM) (Figure 4b,c), similar to the empty control nanoparticles (NPs‐aCD11b). In addition, JT@NPs‐aCD11b had a particle size of approximately 120 nm (Figure 4d), which was similar to that of JQ1@NPs‐aCD11b and Try@NPs‐aCD11b. Notably, the surface charge of JT@NPs‐aCD11b was significantly lower than that of JQ1@NPs‐aCD11b and Try@NPs‐aCD11b (≈−16.4 mV), demonstrating the successful co‐loading of Try and JQ1 (Figure 4e). The successful coupling of CD11b antibodies to the nanoparticles was confirmed by circular dichroism spectroscopy (CDs), which displayed a characteristic peak of the antibody protein in the JT@NPs‐aCD11b formulation (Figure 4f). Additionally, JQ1@NPs‐aCD11b, Try@NPs‐aCD11b, and JT@NPs‐aCD11b achieved similar loading capacities (LC) (≈8.6 wt.%). The encapsulation efficiencies of Try and JQ1 in JT@NPs‑aCD11b were 83.2 ± 3.5% and 86.7 ± 2.9%, respectively, indicating highly efficient co‑encapsulation. Stability assessments in neutral phosphate‐buffered saline (PBS) and in PBS containing 10% fetal bovine serum (FBS) at 37°C showed no significant change in the size of JT@NPs‐aCD11b (Figure 4g and Figure S6). Under mildly acidic conditions, the SM102 component in JT@NPs‐aCD11b becomes protonated, gaining a positive charge. This leads to charge repulsion and a proton sponge effect, destabilizing the lipid nanoparticle structure and triggering its disruption, which promotes rapid release of the encapsulated drug (Figure 4h). As shown in the release kinetics profile (Figure 4i,j), JT@NPs‐aCD11b exhibited strong pH dependence. After 8 h of incubation, the release of Try and JQ1 remained low at pH 7.4 (≈5% and ≈10%, respectively), but was significantly enhanced at pH 5.5 (∼20% and ≈40%, respectively). After 48 h, the cumulative release in the neutral environment was only ≈15%, whereas it increased dramatically to ≈80% in the acidic environment.

**FIGURE 4:**
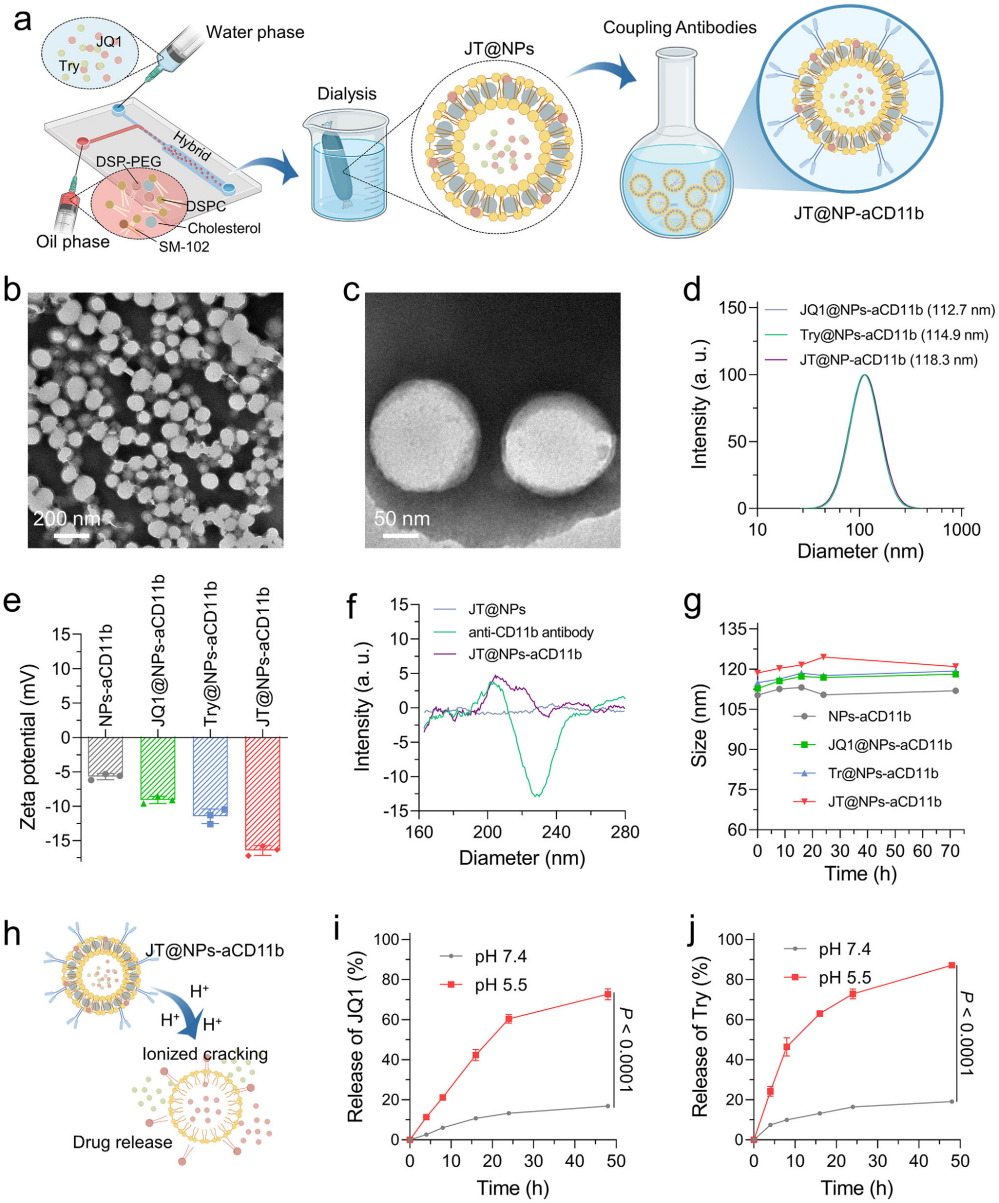
Preparation and physicochemical characterization of JT@NPs‐aCD11b. (a) Formulation schematic of JT@NPs‐aCD11b. TEM images of (b) JT@NPs and (c) JT@NPs‐aCD11b. (d) The size distribution of JQ1@NPs‐aCD11b, Try@NPs‐aCD11b, and JT@NPs‐aCD11b. (e) The charge of different samples (*n* = 3). (f) The CDs of JT@NPs, anti‐CD11b antibody, and JT@NPs‐aCD11b. (g) The change in size of different samples incubated in PBS over 70 h. (h) Schematic diagram of the drug release from JT@NPs‐aCD11b through ionization under mildly acidic conditions The in vitro (i) JQ1 and (j) Try release from JT@NPs‐aCD11b when incubated in release medium (pH 5.5 and 7.4) (*n* = 3; ***p< 0.0001).

### 2.4 Ex Vivo Studies of JT@NPs‐aCD11b

To evaluate the macrophage‐targeting efficacy of JT@NPs‐aCD11b, we performed flow cytometry and confocal laser scanning microscopy (CLSM) on bone marrow‐derived macrophages (BMDMs). Flow cytometry revealed that JT@NPs‐aCD11b was internalized by macrophages significantly more than PBS and the non‐targeted JT@NPs (Figure 5a and Figure S7). This uptake was further confirmed using CLSM, which showed that JT@NPs‐aCD11b exhibited significant internalization after 4 h of co‐incubation with macrophages (Figure 5b and Figure S8). Collectively, these results demonstrate the excellent macrophage‐targeting capability of JT@NPs‐aCD11b. Subsequently, the efficacy of JT@NPs‐aCD11b in promoting macrophage polarization was evaluated in M2 macrophages. Compared to PBS and JQ1@NPs‐aCD11b, both Try@NPs‐aCD11b and JT@NPs‐aCD11b significantly activated the NF‐κB pathway, as evidenced by increased levels of NLRP3, IL‐1β, caspase‐1, and phosphorylated NF‐κB p65 (Figure 5c and Figure S9). Following NF‐κB activation, JT@NPs‐aCD11b treatment led to significant downregulation of M2‐associated markers (Arg1, Fizz1, TGF‐β, and IL‐10) (Figure 5d), while upregulating M1‐associated markers (IL‐1β, TNF‐α, iNOS, and IL‐12) (Figure 5e). Flow cytometry further confirmed the shift toward M1 polarization, showing an increased proportion of F4/80^+^CD86^+^ macrophages and a decreased proportion of F4/80^+^CD206^+^ macrophages after treatment with Try@NPs‐aCD11b and JT@NPs‐aCD11b (Figure 5f,g and Figure S10). Moreover, CLSM was used to assess macrophage phagocytic capacity toward tumor cells. Both JQ1@NPs‐aCD11b and Try@NPs‐aCD11b enhanced macrophage phagocytosis compared to the control, with JT@NPs‐aCD11b inducing the most potent phagocytic activity, attributed to the synergistic induction of M1 polarization and suppression of CD47 in tumor cells (Figure 5h and Figure S11). As shown in Figure S12, both JQ1@NPs‐aCD11b and JT@NPs‐aCD11b treatment markedly decreased CD47 surface expression on Panc02 cells, supporting that JQ1 downregulates the “don't eat me” signal via transcriptional inhibition. Collectively, these results demonstrate that JT@NPs‐aCD11b effectively delivers Try to promote M2‐to‐M1 repolarization and JQ1 to block CD47‐mediated immune evasion, highlighting its potential for combinational immunotherapy.

**FIGURE 5:**
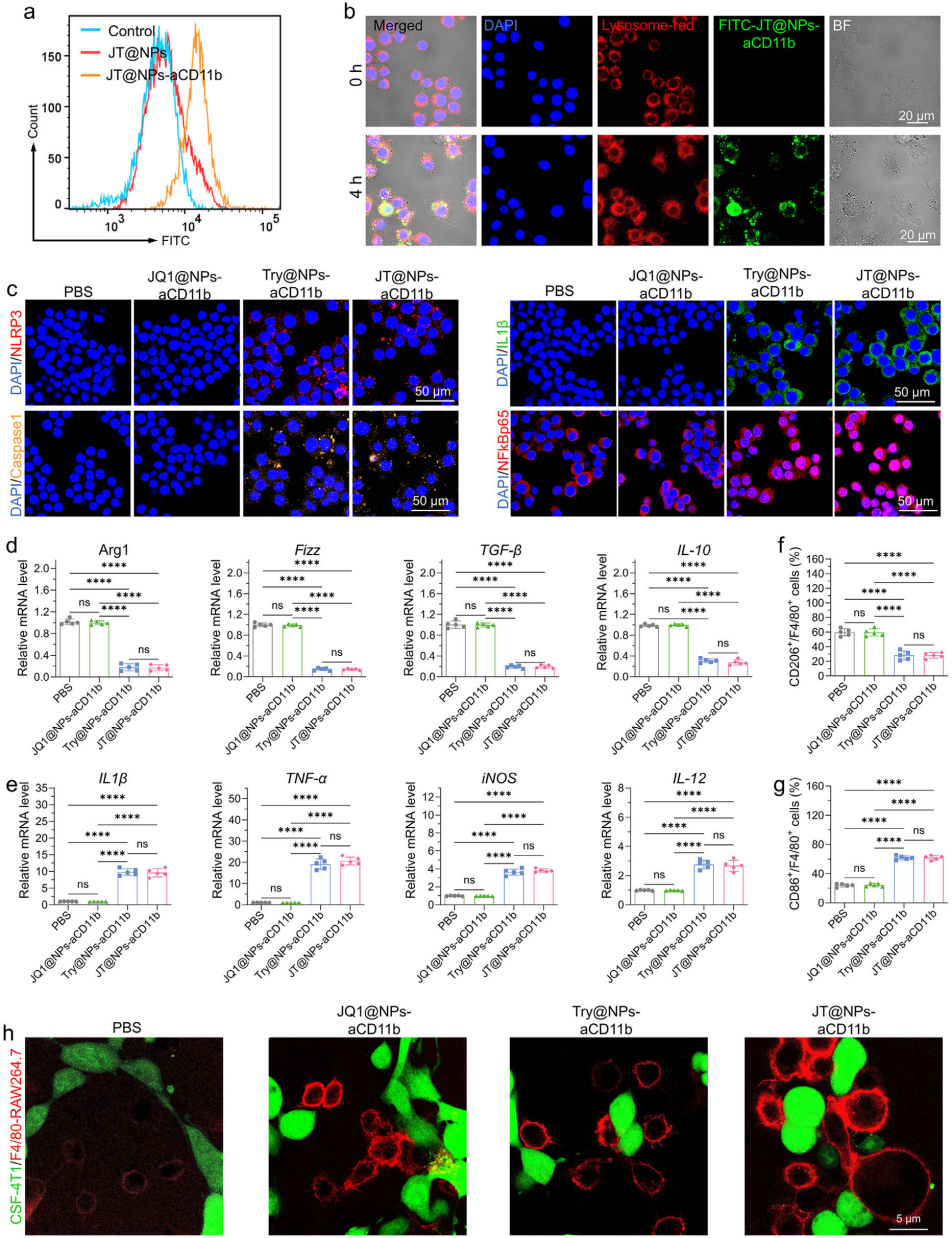
JT@NPs‐aCD11b induces the polarization of M2 macrophages to M1 ones. (a) The cellular uptake of FITC‐labeled JT@NPs‐aCD11b and CD11b‐modified JT@NPs‐aCD11b (both at 50 µM drug loading) was evaluated by flow cytometry (*n* = 3). The quantification was shown in Figure S4. (b) CLSM was used to visualize the internalization of FITC‐labeled JT@NPs‐aCD11b. The quantification was shown in Figure S5. (c) Immunofluorescence staining was performed to detect the expression of NLRP3, Caspase1, IL‐1β, and phosphorylated NF‐κB p65 in M2 macrophages under various treatment conditions. The quantification was shown in Figure S6. RT‐qPCR was conducted to measure mRNA levels of (d) M2‐related genes and (e) M1‐associated genes in treated M2 macrophages (*n* = 5). Flow cytometric quantification of (f) the proportion of F4/80^+^/CD206^+^ macrophages and (g) the proportion of F4/80^+^/CD86^+^ macrophages (*n* = 5). (h) Representative CLSM images show CFSE‐labeled Panc02 tumor cells (green) being phagocytosed by PE‐anti‐CD11b‐stained bone marrow‐derived macrophages (red) following different treatments. Statistical significance was calculated via One‐way Analysis of Variance. Data were expressed as means ± SD in (d–g). ns, no significant, *p< 0.05, **p< 0.01, ***p< 0.001, ****p< 0.0001.

### 2.5 Toxicity, Pharmacokinetics, and Biodistribution of JT@NPs‐aCD11b

The in vivo biocompatibility of JT@NPs‐aCD11b was evaluated in healthy mice (n = 6). Repeated intravenous administration of JT@NPs‐aCD11b (100 mM DL) did not induce significant body weight loss compared to the PBS and free NPs‐aCD11b groups (Figure 6a). Furthermore, liver and kidney function markers were measured to assess potential systemic toxicity. Serum levels of alanine aminotransferase (ALT), aspartate aminotransferase (AST), blood urea nitrogen (BUN), and creatinine (CRE) showed no significant elevation in mice treated with JT@NPs‐aCD11b or free NPs‐aCD11b compared to the PBS control (Figure 6b). Histopathological examination also revealed no obvious morphological damage in major organs, including the heart, liver, spleen, lungs, and kidneys, following treatment with either JT@NPs‐aCD11b or free NPs‐aCD11b (Figure 6c). Serum levels of IL‑6 and TNF‑α in tumor‑bearing mice were also measured by ELISA on days 7, 14, and 21 after treatment with PBS, NPs‑aCD11b, or JT@NPs‑aCD11b (Figure S13). Compared with the PBS control group, JT@NPs‑aCD11b treatment did not cause statistically significant elevations in either cytokine at any time point, and all values remained within the normal physiological range reported for mice. Collectively, these findings support the favorable safety profile of the nanoformulation for in vivo applications. Subsequently, the half‐life of free Try and the JT@NPs‐aCD11b nanoformulation was evaluated in Panc2 tumor‐bearing mice (Figure 6d). The results indicated that the blood circulation time of Try encapsulated in JT@NPs‐aCD11b was significantly prolonged, with a half‐life (t_1_/_2_) of approximately 11 h, compared to about 2 h for free Try (Figure 6d). The biodistribution of JT@NPs‐aCD11b was also assessed in the same model (Figure 6e). To visualize the in vivo targeting efficacy, rhodamine‐labeled nanoformulations were administered intravenously and monitored using an in vivo imaging system (IVIS) at various time points. Imaging of major organs and tumors at 24 h post‐injection revealed significantly stronger fluorescence signals in tumors from mice treated with JT@NPs‐aCD11b compared to those receiving free Try, confirming enhanced tumor accumulation of the nanoformulation (Figure 6f). Furthermore, CLSM images of tumor tissue demonstrated a markedly higher level of JT@NPs‐aCD11b within TAMs relative to free Try (Figure 6g). The superior tumor‐targeting capability of JT@NPs‐aCD11b was further validated in an orthotopic pancreatic cancer model derived from Panc02 cells (Figure 6h and Figure S14). Collectively, these findings demonstrate that the JT@NPs‐aCD11b nanoformulation exhibits improved blood circulation, tumor homing, and TAM‐targeting properties.

**FIGURE 6:**
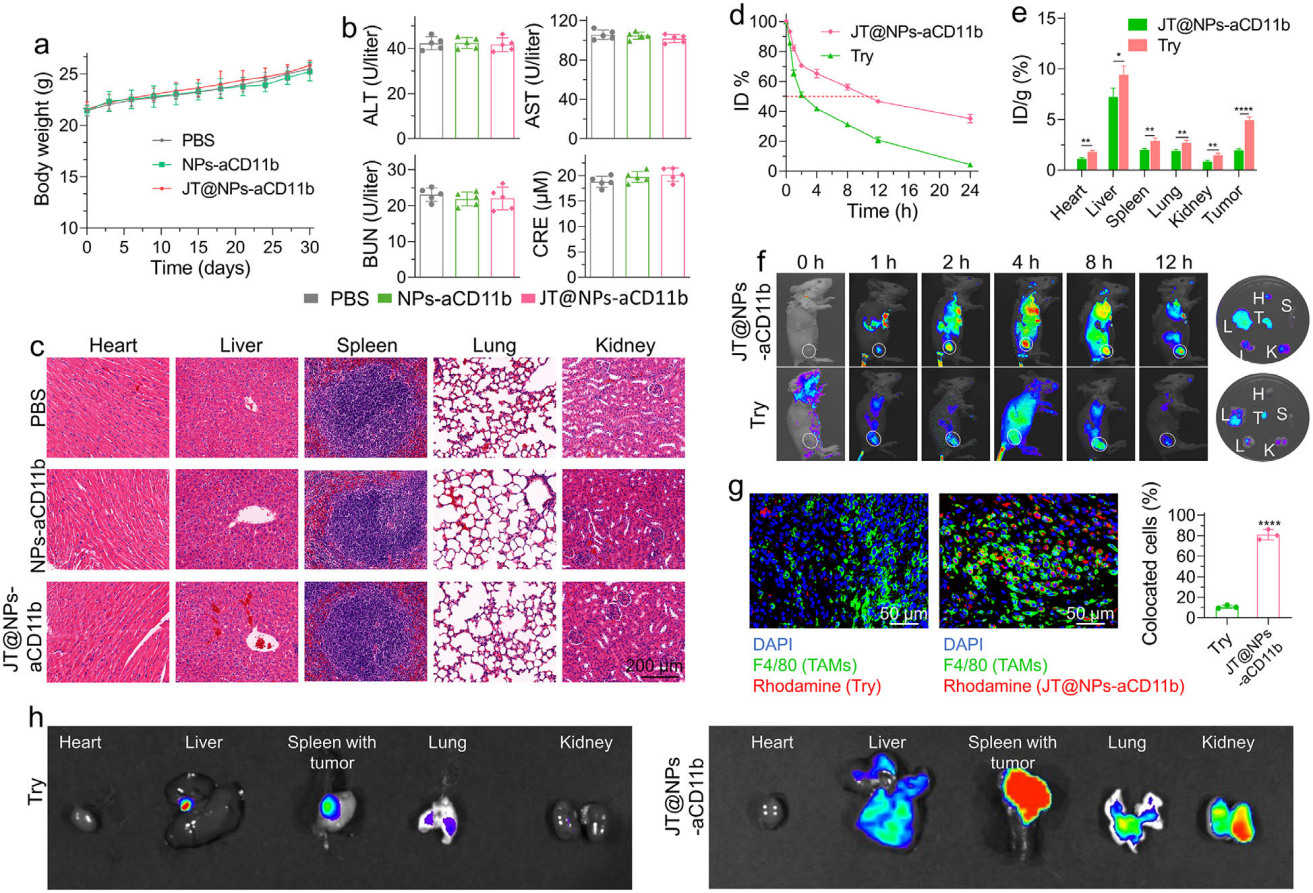
Toxicity, half‐life, and biodistribution of JT@NPs‐aCD11b. (a) Body weight changes in healthy mice over 30 days after intravenous administration (*n* = 5). (b) Liver and kidney function markers‐alanine aminotransferase (ALT), aspartate aminotransferase (AST), blood urea nitrogen (BUN), and creatinine (CRE)‐were measured on day 30 post‐treatment (*n* = 5). (c) Histopathological evaluation of major organs by hematoxylin and eosin (H&E) staining on day 30 after intravenous injection. (d,e) Pharmacokinetic profile of the injected drug concentration (%ID) over time in tumor‐bearing mice (*n* = 3). (f) Biodistribution of rhodamine‐labeled nanoformulations in major organs and tumors at various time points following intravenous injection in subcutaneous tumor‐bearing mice (*n* = 3). (g) Immunofluorescence staining of tumor sections showing rhodamine‐labeled JT@NPs‐aCD11b within TAMs after the above biodistribution study (blue: nuclei; green: F4/80^+^ cells; red: rhodamine). (h) Organ and pancreatic tumor distribution of rhodamine‐labeled nanoformulations at 12 h post‐injection in orthotopic PANC mice (*n* = 3). Statistical significance was calculated via two independent samples unpaired Student's t‐test. Data were expressed as means ± SD in (e, g). ns, no significant, *p< 0.05, **p< 0.01, ***p< 0.001, ****p< 0.0001.

### 2.6 Polarization of TAMs and Remodeling of ITME by Jt@NPs‐aCD11b in PANC

To test the hypothesis that Try repolarizes M2‐like TAMs toward an M1 phenotype and remodels the TME in PANC, we first evaluated its therapeutic efficacy in a Panc02 tumor‐bearing mouse model (Figure 7a). The results showed that JQ1@NPs‐aCD11b alone did not significantly inhibit tumor growth compared to PBS, which is consistent with the low immunogenicity of PANC (Figure 7b–d). In contrast, Try@NPs‐aCD11b significantly suppressed tumor progression relative to PBS or JQ1@NPs‐aCD11b. Notably, JT@NPs‐aCD11b elicited the strongest tumor growth inhibition among all groups over the 21‐day period. Accordingly, treatment with JT@NPs‐aCD11b markedly prolonged animal survival (all mice survived beyond 72 days) compared to PBS (median survival ≈ 35 days), JQ1@NPs‐aCD11b (median survival ≈ 36 days), and Try@NPs‐aCD11b (median survival ≈ 52 days) (Figure S15). Importantly, macrophage depletion via clodronate liposomes (CL) abrogated the anti‐tumor effect of JT@NPs‐aCD11b, indicating that its efficacy primarily depends on macrophage engagement and activation rather than on other immune cells. Subsequently, the efficacy of JT@NPs‐aCD11b in macrophage modulation for anti‐PANC therapy was confirmed. Immunofluorescence staining revealed that JT@NPs‐aCD11b markedly reduced the proportion of F4/80^+^Arg1^+^ M2‐type TAMs to approximately 3%, compared with PBS (≈19%), JQ1@NPs‐aCD11b (≈16%), and Try@NPs‐aCD11b (≈8%) (Figure S16). In contrast, JT@NPs‐aCD11b significantly increased the infiltration of F4/80^+^iNOS^+^ M1 macrophages (≈20%) within tumors relative to other groups. In the JT@NPs‐aCD11b + CL group, minimal F4/80^+^ macrophages were detected due to the depletion effect of CL. Following TAM polarization, we evaluated the ability of JT@NPs‐aCD11b to remodel the ITME. Flow cytometry revealed a marked reduction in immunosuppressive cells—including MDSCs and Tregs—in tumors treated with Try@NPs‐aCD11b and JT@NPs‐aCD11b, compared to PBS, JQ1@NPs‐aCD11b, and JT@NPs‐aCD11b + CL groups (Figure 7e,f). Conversely, immunostimulatory populations such as NK cells, activated dendritic cells (DCs), cytotoxic and memory CD8^+^ T cells, and helper and memory CD4^+^ T cells were substantially increased following treatment with Try@NPs‐aCD11b or JT@NPs‐aCD11b (Figure 7g–l and Figure S17). Consistent with these cellular shifts, JT@NPs‐aCD11b administration led to decreased levels of immunosuppressive cytokines, including IL‐10, TGF‐β, and IL‐4 (Figure S18), while elevating immunostimulatory factors such as IFN‐γ, TNF‐α, and IL‐12 (Figure S18). Immunohistochemical staining showed that Try@NPs‐aCD11b and JT@NPs‑aCD11b treatment significantly increased the expression of calreticulin (CRT) and heat shock protein 70 (HSP70), and release of high mobility group box 1 (HMGB1), in tumor tissues compared with other groups (Figure S19), indicating efficient induction of immunogenic cell death (ICD). These DAMP changes correlated with enhanced DC maturation and augmented T‑cell effector functions, supporting that JT@NPs‑aCD11b triggers a cascade from tumor cell killing to adaptive antitumor immunity. This remodeling of the TME further induced significant tumor apoptosis (≈72%) in the JT@NPs‐aCD11b group, outperforming PBS (≈2%) and JQ1@NPs‐aCD11b (≈7%) treatments (Figure S20). As a key indicator of adaptive immune activation, the presence of CD4^+^ and CD8^+^ memory T cells—capable of conferring long‐term protection against tumor rechallenge—was notably elevated in JT@NPs‐aCD11b‐treated mice. To assess whether this response translated into durable immunity, we rechallenged cured mice with Panc02 cells in the opposite flank 40 days after the initial treatment (Figure 7m). While all control mice developed tumors, tumor growth was strongly suppressed in the JT@NPs‐aCD11b group (Figure 7n,o). Moreover, all treated mice survived beyond 100 days post‐rechallenge (Figure S21). These findings underscore the capacity of JT@NPs‐aCD11b to reverse immunosuppression in the TME and elicit robust anti‐tumor immunity, primarily mediated through TAM reprogramming (Figure 7).

**FIGURE 7:**
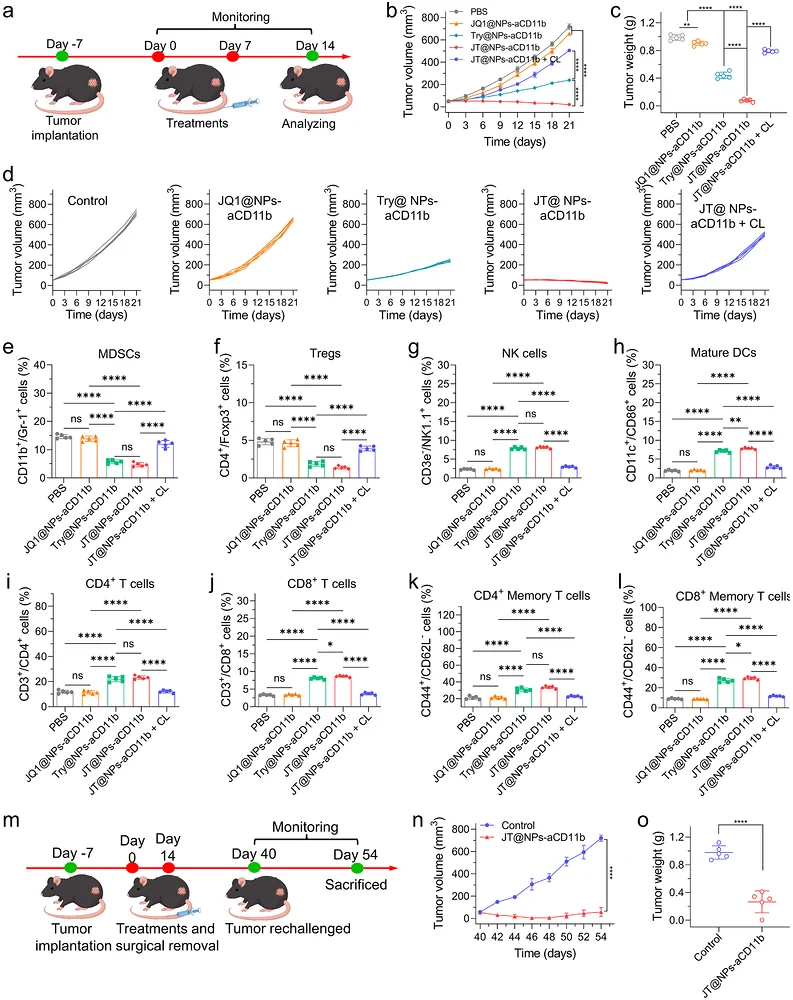
JT@NPs achieves antitumor effects by polarizing TAMs from M2 to M1 and remodeling ITME in PANC. (a) Establishment of model mice and treatment scheme. (b) The tumor development over a 21‐day period (*n* = 5). (c) The tumor weight of PANC mice at the end of different treatments (*n* = 5). (d) Individual tumor growth curves for different groups. The quantification was shown in Figure S14. The quantification of immunosuppressive cells such as (e) MDSCs and (f) Tregs and immunostimulatory cells such as (g) NK cells, (h) activated dendritic cells (DCs), (i, j) CD8^+^ cytotoxic and memory T cells, and (k, l) CD4+ helper and memory T cells in tumors derived from different groups analyzed by flow cytometry (*n* = 5). (m) Schematic diagram of the experimental design for analyzing immune memory effects in cured mice following various treatments. (n) The average volume changes of rechallenged tumors across different treatment groups (*n* = 5). (o) Individual tumor volume trajectories for each mouse are presented. Statistical significance was calculated via One‐way Analysis of Variance. Data were expressed as means ± SD in (c, e‐l, n, o). ns, no significant, *p< 0.05, **p< 0.01, ***p< 0.001, ****p< 0.0001.

### 2.7 Systemic Dose of JT@NPs‐aCD11b Reduces Orthotopic Pancreatic Tumor Burden

As demonstrated above, JT@NPs‐aCD11b promoted the repolarization of TAMs from the M2 to the M1 phenotype and remodeled the ITME in PANC. To more closely mimic the in vivo tumor immune microenvironment, we further assessed the antitumor efficacy of JT@NPs‐aCD11b using an orthotopic PANC model established with Panc02‐luc cells (Figure 8a). Mice bearing orthotopic Panc02‐luc‐derived tumors were randomly divided into five treatment groups: PBS (control), JQ1@NPs‐aCD11b, Try@NPs‐aCD11b, JT@NPs‐aCD11b, and JT@NPs‐aCD11b + CL. Treatments were administered intravenously every four days, and tumor growth was monitored via in vivo imaging. Imaging results revealed that JQ1@NPs‐aCD11b did not significantly inhibit tumor growth compared to the PBS group, whereas Try@NPs‐aCD11b modestly delayed tumor progression (Figure 8b,c). In contrast, JT@NPs‐aCD11b treatment resulted in marked suppression of tumor growth over the 23‐day observation period, compared to either JQ1@NPs‐aCD11b or Try@NPs‐aCD11b alone (Figure 8b,c). Furthermore, depletion of macrophages using CL abolished the therapeutic benefit of JT@NPs‐aCD11b. Survival analysis showed that JT@NPs‐aCD11b treatment led to prolonged survival, with 5 out of 10 mice surviving beyond 50 days, compared to median survival times of approximately 25 days in the PBS group, 26 days with JQ1@NPs‐aCD11b, 32 days with Try@NPs‐aCD11b, and 29 days with JT@NPs‐aCD11b + CL (Figure 8d).

**FIGURE 8:**
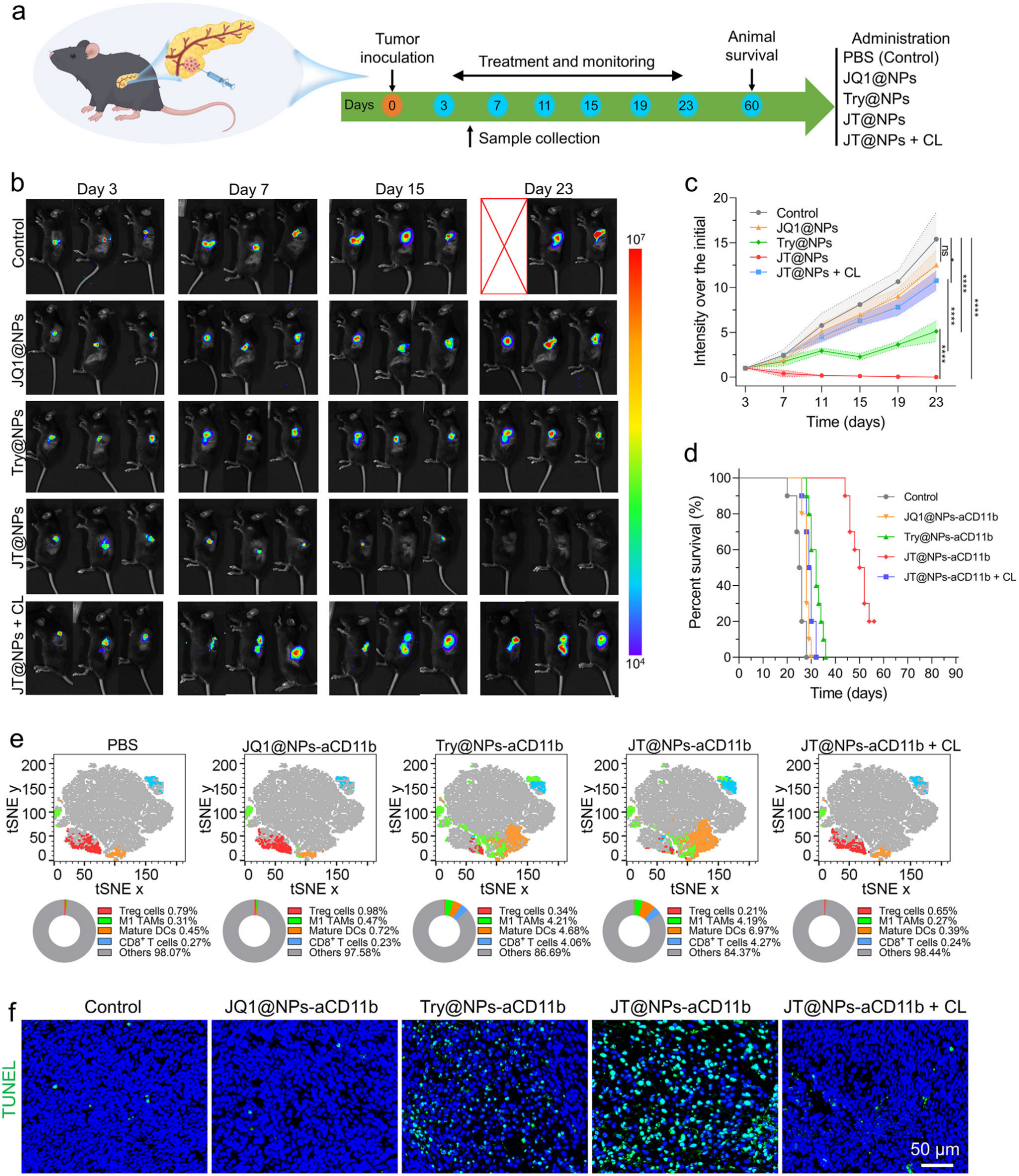
JT@NPs‐aCD11b achieves antitumor effects in Panc02‐luc‐derived orthotopic PANC. (a) Establishment of orthotopic PANC model mice and treatment scheme. (b) The representative in vivo imaging images of pan02‐luc‐derived orthotopic PANC mice with different treatments (*n* = 3). (c) The PANC development over a 23‐day period (*n* = 3). (d) Survival in the PANC mice model after different treatments (*n* = 10). (e) tSNE visualization and quantification of different immune cells in tumors by cytometry from orthotopic PANC tumor‐bearing mice after different treatments. (f) Immunohistochemical staining images of the TUNEL nick‐end labeling assay of tumor tissue from different groups. The quantification was shown in Figure S19. Statistical significance was calculated via two independent samples unpaired Student's t‐test. Data were expressed as means ± SD in (c, d). ns, no significant, *p< 0.05, **p< 0.01, ***p< 0.001, ****p< 0.0001.

We subsequently assessed immune cell infiltration in mouse tumors on day three following different treatments using multicolor flow cytometry. As expected, neither JQ1@NPs‐aCD11b nor JT@NPs‐aCD11b + CL injection induced notable changes in intratumoral immune cell populations—such as Tregs, M1 TAMs, matured DCs, and CD8^+^ T cells—compared to PBS injection, reflecting a tumor immune “cold” state (Figure 8e). In contrast, treatment with Try@NPs‐aCD11b significantly increased the infiltration of M1 TAMs, matured DCs, and CD8^+^ T cells to 4.19%, 6.97%, and 4.27%, respectively, compared to 0.31%, 0.45%, and 0.27% in the control group, while simultaneously suppressing Tregs infiltration. Notably, JT@NPs‐aCD11b treatment further enhanced immune cell recruitment into tumors, attributable to the additional effect of CD47 inhibition. Immunofluorescence staining (Figure S22) revealed that Try@NPs‐aCD11b significantly reduced the infiltration of M2‐type tumor‐associated macrophages (F4/80^+^/Arg1^+^) within tumors compared to other groups. The co‐delivery of JQ1 and the tyrosine kinase inhibitor via JT@NPs‐aCD11b further substantially decreased M2 TAM populations. Furthermore, JT@NPs‐aCD11b treatment most potently elevated the number of M1‐type macrophages (F4/80^+^/iNOS^+^). This shift in macrophage polarization indicated that our combinatorial strategy effectively reprogrammed the immunosuppressive tumor microenvironment into an immunostimulatory one, which was corroborated by a concomitant decrease in myeloid‐derived suppressor cells and regulatory T cells, alongside an increase in NK cells, activated dendritic cells, and effector T cells (Figure S23). Consistent with this immune remodeling, the JT@NPs‐aCD11b regimen induced the most significant level of tumor cell apoptosis, outperforming the PBS control, individual drug‐loaded nanoparticles, and the clearance control group (Figure 8f and Figure S24). These findings collectively demonstrate that our nanoformulation can modulate macrophage polarization to remodel the immunosuppressive tumor microenvironment, significantly enhancing therapeutic efficacy against orthotopic pancreatic cancer and presenting a promising combinatorial treatment approach.

### 2.8 Single‐Cell RNA Sequence Analysis Identifies Altered Cell Composition in PANC

To evaluate the therapeutic efficacy of JT@NPs‐aCD11b, we conducted single‐cell RNA sequencing (scRNA‐seq) on tumor tissues obtained from a PANC mouse model following treatment with JT@NPs‐aCD11b. Using the Seurat package in R, we performed unsupervised clustering on an equivalent number of viable cells isolated from three control tumors and three JT@NPs‐aCD11b‐treated tumors. Uniform Manifold Approximation and Projection (UMAP) visualization, guided by canonical lineage markers, identified 12 major cell populations across all samples (Figure 9a,b). Comparative UMAP visualization and quantitative assessment revealed that treatment with JT@NPs‐aCD11b led to a notable increase in the infiltration of immune effector cells—such as macrophages, dendritic cells, T cells, and natural killer (NK) cells—relative to the control group (Figure 9c,d). Among these, macrophages exhibited the most substantial increase. To further delineate the functional role of activated macrophages, we analyzed macrophage subpopulations in both control and JT@NPs‐aCD11b‐treated tumors. Subclustering analysis based on UMAP identified five principal macrophage subtypes: Crr2^+^, Mmp12^+^, Cdl8^+^, Pclaf1^+^, and Fabp7^+^ (Figure 9e,f). In the JT@NPs‐aCD11b group, a marked elevation in Crr2^+^ macrophages was observed—a subtype associated with high M1‐polarization scores and upregulated pro‐inflammatory pathway activity. Conversely, Fabp7^+^ macrophages, which exhibit strong M2‐polarization signatures and diminished pro‐inflammatory gene expression, were significantly reduced (Figure 9g–i). Cell–cell communication analysis performed with the CellChat algorithm indicated strengthened interactions between tumor‐associated macrophages (TAMs) and other immune cells in JT@NPs‐aCD11b‐treated tumors (Figure 9j), suggesting facilitation of adaptive immune activation. Specifically, CD86 and ICAM signaling between TAMs and T cells was considerably enhanced upon JT@NPs‐aCD11b treatment (Figure 9k), while TGF‐β signaling was notably suppressed—a pattern indicative of robust T cell activation. Collectively, these findings indicate that JT@NPs‐aCD11b induces macrophage repolarization toward an anti‐tumor phenotype through pro‐inflammatory pathway activation, reverses the ITME, and enhances adaptive immune responses, ultimately inducing potent anti‐tumor immunity.

**FIGURE 9:**
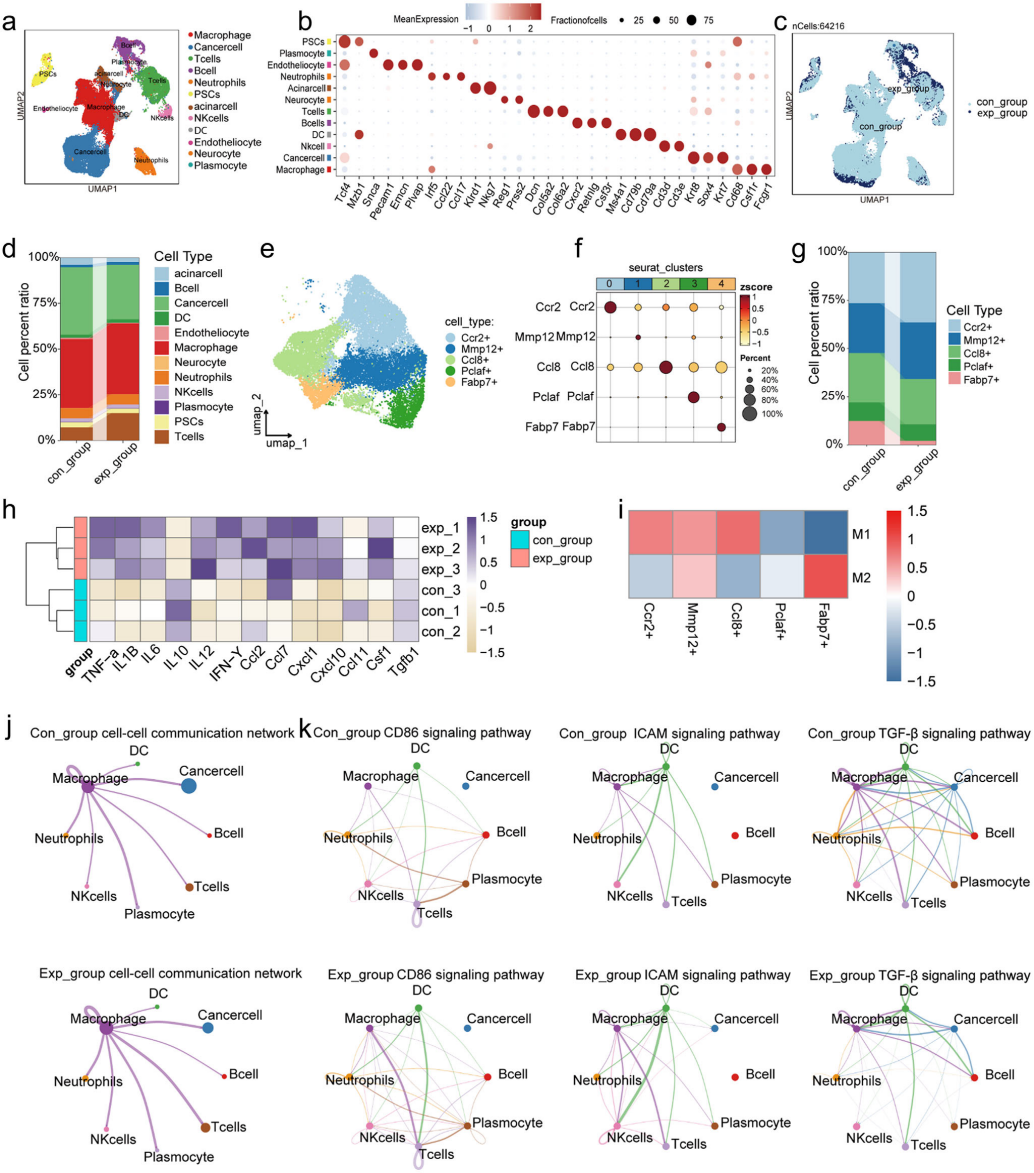
Single‐cell resolution sequencing analysis of tumor tissue transcriptomes obtained from PANC model mice following JT@NPs‐aCD11b treatment. (a) UMAP visualization of the 12 main cell types presented in tumor tissues from PANC model mice (*n* = 3). (b) Key marker genes defining each type of cell cluster in (a) are presented. (c) UMAP visualization of cell compositions of indicated immune cell subpopulations in control and JT@NPs‐aCD11b groups. (d) The relative abundance of indicated cell populations in control and JT@NPs‐aCD11b groups. (e) UMAP visualization of cell compositions of indicated macrophage subpopulations. (f) The signature genes of defined macrophage subpopulations are shown in a dot plot. (g) The relative abundance of indicated macrophage subpopulations in control and JT@NPs‐aCD11b group. (h) Heat map of M2‐ type and M1‐type macrophages‐related gene expression levels in control and JT@NPs‐aCD11b groups. (i) Scores heat map of macrophage polarization gene expression in different macrophage subpopulations. (j) The interaction network diagram between macrophages and other major immune cells in the JT@NPs‐aCD11b group and the control group at the overall level and different pathway levels, based on the “CellChat” algorithm. Line thickness represents interaction strength. Dot size represents the cell number of the indicated cell subpopulation.

## 3 Discussion

PANC remains a formidable malignancy characterized by a profoundly ITME, which is predominantly orchestrated by TAMs [28, 29, 30]. The high density of M2‐polarized TAMs strongly correlates with disease progression, immune evasion, and resistance to conventional therapies [31]. Consequently, reprogramming protumoral M2‐TAMs toward an antitumoral M1 phenotype has emerged as a promising therapeutic strategy [32, 33, 34]. Our study identifies Try as a novel and potent inducer of M1 macrophage polarization. This discovery originated from the paradoxical observation that Try, while known for its detrimental role in acute pancreatitis by triggering pro‐inflammatory cascades within phagocytic macrophages, shares a cytokine activation profile reminiscent of M1 polarization. We subsequently validated that Try effectively reprograms M2 macrophages to an M1‐like state, as evidenced by the downregulation of M2 markers and the concurrent upregulation of M1 markers. Mechanistically, we demonstrated that this polarization shift is primarily mediated through the activation of the canonical NF‐κB signaling pathway, specifically via phosphorylation of the p65 subunit at serine 536, leading to its nuclear translocation and the initiation of a pro‐inflammatory gene transcription program. This delineation of a specific molecular pathway not only provides a solid foundation for Try's immunomodulatory function but also distinguishes it from other polarization agents. To overcome the inherent limitations of Try for systemic administration—such as rapid clearance, lack of tumor specificity, and potential toxicity—we engineered a targeted “smart” nanoresponder, JT@NPs‐aCD11b. This ionizable liposomal nanoparticle was designed for the co‐delivery of Try and the CD47‐SIRPα checkpoint inhibitor JQ1, with surface functionalization of CD11b antibodies ensuring specific targeting toward TAMs. The rational combination within this nano‐platform is critical: while Try drives the M2‐to‐M1 repolarization, JQ1 blocks the “don't eat me” signal on tumor cells, thereby synergistically enhancing the phagocytic capacity of the newly polarized M1 macrophages. Our comprehensive in vitro and in vivo characterizations confirm the successful fabrication, excellent stability, pH‐responsive drug release, efficient cellular uptake, and favorable safety profile of JT@NPs‐aCD11b, underscoring its potential as a reliable delivery system for combinatorial immunotherapy.

The therapeutic efficacy of JT@NPs‐aCD11b was rigorously evaluated in both subcutaneous and orthotopic murine models of pancreatic cancer. The nanoformulation demonstrated superior tumor growth suppression and significantly prolonged host survival compared to control groups or monotherapy formulations. A key finding was that the anti‐tumor effect was abrogated upon macrophage depletion, unequivocally establishing the central role of TAMs in mediating the therapeutic outcome. Our data reveal a multi‐faceted mechanism of action. First, JT@NPs‐aCD11b effectively reshaped the cellular composition of the TME. It significantly increased the ratio of M1 to M2 TAMs within tumors, as confirmed by flow cytometry, immunofluorescence, and single‐cell RNA sequencing (scRNA‐seq). scRNA‐seq further delineated a marked increase in the Crr2^+^ macrophage subpopulation (enriched with M1 signatures) and a decrease in the Fabp7^+^ subpopulation (associated with M2 phenotypes). Second, this TAM reprogramming initiated a cascade of immunomodulatory events. We observed a substantial reduction in immunosuppressive cells, including MDSCs and Tregs, and a concomitant influx of immune effector cells, such as activated dendritic cells, natural killer (NK) cells, and cytotoxic CD8^+^ T cells. Cell–cell communication analysis via the CellChat algorithm further revealed enhanced interactions, particularly through CD86 and ICAM signaling between TAMs and T cells, alongside attenuated TGF‐β signaling, indicative of a robustly activated adaptive immune response. Finally, this remodeled, immunologically “hot” TME not only induced significant tumor cell apoptosis but also established a potent and durable anti‐tumor memory, effectively protecting cured mice from tumor rechallenge. This transition from a suppressive to a permissive immune milieu highlights the potential of our strategy to overcome the hallmark immunosuppression of PANC.

Despite these promising results, several challenges remain before clinical translation can be realized. The long‐term biocompatibility and potential immunogenicity of the nanocarrier components require further investigation in higher‐order animal models. Additionally, the scalability and manufacturing reproducibility of the targeted nanoformulation under Good Manufacturing Practice standards present significant translational hurdles.

## 4 Conclusions

Drawing inspiration from the pathogenesis of pancreatitis, we have discovered that Try is a novel and effective immunomodulator capable of reprogramming M2 macrophages into the M1 phenotype—which possesses antitumor activity—by activating the NF‐κB pathway. Based on this finding, we engineered a sophisticated smart nanoplatform, JT@NPs‐aCD11b, for the targeted co‐delivery of Try and the CD47‐SIRPα checkpoint inhibitor JQ1. This combinatorial nano‐strategy demonstrates remarkable synergistic efficacy: Try effectively repolarizes TAMs, while JQ1 concurrently blocks the “don't eat me” signal on cancer cells, thereby enhancing macrophage‐mediated phagocytosis. In a rigorous preclinical model of pancreatic cancer, this dual‐pronged approach effectively reversed the immunosuppressive landscape, characterized by a significant increase in M1 macrophages and cytotoxic T cells, along with a concomitant decrease in immunosuppressive cells. Consequently, treatment with JT@NPs‐aCD11b potently suppressed tumor progression, inhibited metastasis, and markedly extended animal survival. These compelling findings not only validate Try as a powerful TAM‐targeting agent but also position JT@NPs‐aCD11b as a highly promising and translatable immunotherapeutic nanoplatform for recalcitrant malignancies like PANC.

### 4.1 Statistical Analysis

For all experiments, the investigators were blinded to group allocation during data collection and/or analysis. For independent experiments with a sample size N ≥ 3, data were expressed as mean ± standard deviation (SD). Multiple comparisons were conducted using one‐way ANOVA, while differences between two groups were analyzed using two‐way ANOVA. Statistical analyses were performed and displayed using Graphpad Prism 9.5. *p* values less than 0.05 were considered statically significant. *p < 0.05, **p < 0.01, ***p < 0.001, ****p < 0.0001 and ns for non‐significant.

## Author Contributions

**Lei Cao**, Conceptualization: **Yuan Yong**, **Wencheng Wu**; methodology: **Lei Cao**, **Yu He**, **Wenhao Li**, **Wencheng Wu**; Investigation: **Yu He**, **Wenhao Li**, **Han Lin**; visualization: Lei Cao, Wenhao Li, Han Lin; Supervision: Han Lin, **Bo Gong**, Yuan Yong; Writing – original Draft: Lei Cao, Yu He, Wencheng Wu; Writing – review & Editing: **Lei Cao**, **Yu He**, **Wenhao Li**, **Yuan Yong**, **Jianlin Shi**, **Wencheng Wu**.

## Ethics Statement

This research complies with all relevant ethical regulations. Experiments were performed in agreement with the Animal Care Ethics Commission of Sichuan Academy of Medical Sciences, Sichuan Provincial People's Hospital, University of Electronic Science and Technology of China (ID: SYXK‐2023‐279). No human experiments or human tissue samples were involved in this study.

## Conflicts of Interest

The authors declare no conflicts of interest.

## Supporting information




**Supporting File**: advs76386‐sup‐0001‐SuppMat.docx.

## Data Availability

The data that support the findings of this study are available from the corresponding author upon reasonable request.
