# Supplementary material for: A Pancreatitis‐Inspired Trypsinogen Nanoplatform Reprograms Tumor‐Associated Macrophages via NF‐κB for Pancreatic Cancer Immunotherapy
Source: Adv Sci (Weinh). 2026 Jun 27;13(54):e76386. doi: 10.1002/advs.76386 (PMC13336749; doi:10.1002/advs.76386)
Supplement: Supplementary file 1 [file ADVS-13-e76386-s001.docx]

***Supporting Information for***

**A Pancreatitis-Inspired Trypsinogen Nanoplatform Reprograms Tumor-Associated Macrophages via NF-κB for Pancreatic Cancer Immunotherapy**

**Materials and methods**

**Chemicals and reagents**

Ionizable lipid (SM102) was purchased from SinoBang (Xiamen) Biological Technology Co., Ltd. 1,2-Dioctadecanoyl-sn-glycero-3-phosphocholine (DSPC) and cholesterol were purchased from AVT (Shanghai) Pharmaceutical Tech Co., Ltd. 1,2-distearoyl-sn-glycero-3-phosphoethanolamine-N-[amino(polyethylene glycol) 2000] (DSPE-PEG-2000) were obtained from Xi’an Ruixi Biological Technology Co. (Xian, China). Trypsinogen (Try) and JQ1 were purchased from Shanghai Macklin Biochemical Co., Ltd.‌ All reagents were used as received without further purification. Phosphate buffer solution (PBS), Dulbecco's modified eagle medium (DMEM), calcein, 4, 6-diamidino-2-phenylindole (DAPI), propidium iodide (PI), fluorescein isothiocyanate (FITC), Superior FBS (U11-020A), and cell counting Kit-8 (CCK-8) were purchased from YOBIBIO (Shanghai, China). All the antibodies used in this work were purchased from BioLegend, Inc.

**Animals**

Female 5- to 6-week-old Babl/c and C57 mice were bought in Beijing Vital River Laboratory Animal Technology Company. Experiments were approved by the Experimental Animal Ethics Committee of Animal Care Ethics Commission of Sichuan Academy of Medical Sciences, Sichuan Provincial People’s Hospital, University of Electronic Science and Technology of China (ID: SYXK-2023-279).

**Cell culture**

Mouse 4T1 breast cells and Mouse Panc-02 cells were cultured in high-glucose Dulbecco’s modified Eagle medium (DMEM; Gibco) supplemented with 10% fetal bovine serum (FBS) and 1% penicillin-streptomycin (Thermo Fisher Scientific). Mouse Panc-02-luc cells (stably expressing luciferase) were cultured in DMEM containing 10% FBS, 1% penicillin-streptomycin, and puromycin (1 μg/ml; Thermo Fisher Scientific). Cells were maintained at 37°C, 5% CO2, and 95% relative humidity.

**Ex vivo characterization of Try**

The preparation of bone marrow-derived macrophages (BMDMs) followed a multi-step process. Firstly, bone marrow cells were harvested from the femurs and tibiae of 5-6-week-old C57 mice. These cells were then differentiated into BMDMs over a 5-day period in RPMI 1640 medium (Corning) enriched with 10% FBS, 1% penicillin-streptomycin, and 20 ng/ml macrophage colony-stimulating factor (Thermo Fisher Scientific). Following the differentiation, the BMDMs were polarized towards M1 or M2 phenotypes by a 48-hour stimulation with either IFN-γ (20 ng/ml) plus LPS (100 ng/ml) or IL-4 (20 ng/ml), respectively. For the final assay, the macrophages were seeded in six-well plates (2 × 10^5 cells/well) and, after overnight adhesion, were incubated with or without 50 mM Try for 24 hours. Morphological assessment was performed by imaging under a light microscope.

Macrophages were plated in six-well plates at a density of 2 × 10⁵ cells per well and treated with varying concentrations of Try (10, 50, and 100 mM) for 24 hours. The percentages of F4/80⁺CD206⁺ (M2) and F4/80⁺CD86⁺ (M1) macrophage subsets were quantified by flow cytometry (BD FACSCalibur), and data analysis was performed using FlowJo software (version 7.6).

In a separate experiment, macrophages exposed to 50 mM Try for 24 hours were subjected to transcriptome analysis. Total RNA was isolated using the RNeasy MiniElute Kit (Qiagen), reverse-transcribed into cDNA, and sequenced on an Illumina platform by Biomarker Technologies Co. Ltd. Differential gene expression was assessed, and gene set enrichment analysis (GSEA) was conducted with a false discovery rate (FDR) q-value threshold of < 1. Functional annotation based on GO and KEGG pathways was carried out via the BMKCloud bioinformatics platform (www.biocloud.net/).

For gene expression validation by quantitative real-time PCR, macrophages were incubated with Try at 10, 50, and 100 mM for 24 hours. RNA was extracted with TRIzol reagent (Invitrogen) and reverse-transcribed using a Reverse Transcription Master Kit (YEASEN Biotech, China). Amplification was performed on a 7500 real-time PCR system (Applied Biosystems) under established conditions, and mRNA expression levels were normalized to glyceraldehyde-3-phosphate dehydrogenase (GAPDH). Additionally, protein expression changes were examined in macrophages treated with 50 mM Try for 24 hours. Whole-cell proteins were extracted and quantified as described previously. For Western blotting, 40 μg of protein per sample was separated, transferred, and probed with specific antibodies. Protein bands were visualized and quantified using previously reported methods.

The immunofluorescence protocol was performed as follows: macrophages were plated at 2 × 10⁵ cells/well in 6-well plates and incubated under various conditions for 24 hours. After the treatment period, the cells were washed with PBS and fixed by a 10-minute exposure to 4% paraformaldehyde. Subsequently, the fixed cells were treated with specific primary antibodies and incubated for 4 h at room temperature. For detection, the cells were stained with FITC- or APC-conjugated secondary antibodies, and the nuclei were labeled with DAPI prior to imaging with a confocal laser scanning microscope.

**Preparation of** **JT@NPs-aCD11b nanoformulation**

JT@NPs were first formulated by microfluidic mixing an aqueous phase containing drugs with an organic phase containing lipid components. The ethanol phase was prepared by dissolving SM102 ionizable lipid, cholesterol, DSPC and DSPE-PEG-2000 at a molar ratio of 50:38.5:10:1.5, respectively in absolute ethanol. The aqueous phase was prepared by dissolving 2 mg/mL JQ1 in 1 mg/mL Try 100 mM citrate buffer at pH 4.0. The aqueous and ethanol phases were rapidly mixed at a volume ratio of 3:1 and a total flow rate of 12 mL/min using a microfluidic device (Micro&Nano). Following dialysis against 20 mM Tris buffer (pH 7.5) for 24 h at 4 °C in a 3.5 kDa MWCO dialysis box (Thermo Fisher, 66330), JT@NPs was harvested by ultrafiltration centrifugation at 3000 × g and 4 °C. The anti-CD11b antibodies were conjugated to the surface of JT@NPs via a classic EDC/NHS amidation coupling reaction. In brief, JT@NPs were first exchanged into MES buffer (pH 6.0), where surface carboxyl groups were activated using EDC and NHS to form amine-reactive NHS esters. After rapid removal of the excess coupling reagents, the activated nanoparticles were incubated with the anti-CD11b antibody in PBS (pH 7.4), allowing the primary amines on the antibody to react with the surface esters to form stable amide bonds. Finally, the product, the targeted nanoformulation JT@NPs-aCD11b, was purified via dialysis for subsequent use.

**Physicochemical and ex vivo characterization of JT@NPs-aCD11b**

The morphological characteristics of the nanoformulations were examined by transmission electron microscopy (TEM; JEM-1230). Particle size, zeta potential, and colloidal stability were measured with a Malvern Nano-ZS analyzer. Drug loading capacity (LC) for Try and JQ1 was quantified via the Colorimetric Assay Kit (BioVision, USA) following the manufacturer’s instructions. LC was calculated as follows: (mass of encapsulated drug / total mass of nanoformulations) × 100%.

The *in vitro* drug release profile of JT@NPs-aCD11b was evaluated using a dialysis method under two different pH conditions (pH 7.4 and 5.5) to simulate the physiological and acidic tumor microenvironments, respectively. The nanoparticle suspension was placed in a dialysis bag and immersed in the corresponding release medium at 37°C under continuous agitation. At predetermined time intervals, samples were withdrawn from the external medium, and the concentrations of released Try and JQ1 were quantified by HPLC.

To evaluate cellular uptake, M2 macrophages were plated in six-well plates at a density of 2 × 10⁵ cells per well and cultured overnight. Cells were then treated with FITC-labeled nanoformulations (0.05 wt% FITC; Try concentration = 50 mM). After 6 h of incubation, the percentage of rhodamine-positive cells was analyzed using flow cytometry.

In addition, M2 macrophages (2 × 10⁵ cells/well) were seeded overnight and exposed to the same nanoformulations (Try = 50 mM). Following 24 h of treatment, NF-κB pathway activation was assessed by Western blot, and the expression levels of key functional markers were determined using quantitative reverse transcription PCR (qRT-PCR). The immunofluorescence procedure is the same as previously described.

**Safety, half-life, and biodistribution of JT@NPs-aCD11b**

The biosafety of the nanoformulations was evaluated in healthy mice (n = 6 per group) receiving intravenous injections of PBS, free NPs-aCD11b (20 mg/kg), or JT@NPs-aCD11b (20 mg/kg). Body weight changes were monitored throughout the study. On day 30, major organs (heart, liver, spleen, lungs, and kidneys) were collected for histopathological examination using hematoxylin and eosin (H&E) staining. Liver and kidney function markers (ALT, AST, BUN, and CRE) were also analyzed at the same time point by Wuhan Servicebio Technology Co. Ltd.

The subcutaneous and orthotopic pancreatic cancer model was established based on a previously described procedure. To establish subcutaneous Panc02 pancreatic tumor models, 5 × 10⁵ Panc02 cells were implanted into the upper surface of the hind leg of female C57 mice. For orthotopic pancreatic cancer model establishment, the spleen was surgically exposed, ligated at the midregion, and divided into two segments, each retaining an intact vascular pedicle. Panc02-luc cells (1 × 10⁶) were then implanted into the pancreas on day 0. Tumor progression was monitored noninvasively using an IVIS imaging system (PerkinElmer) after intraperitoneal administration of 90 μl luciferin (10 μg/μl; Pierce). When the bioluminescence signal reached an intensity between 5 × 10⁸ and 1 × 10⁹ p/s/cm²/sr, the mice were utilized for pharmacokinetic and biodistribution studies.

For pharmacokinetic analysis, mice (n = 4) received intravenous injections of nanoformulations (20 mg/kg), and serum Try levels were measured at various time points using a colorimetric assay kit. For tissue distribution assessment, another group of mice (n = 3) was administered rhodamine-labeled nanoformulations (0.05 wt% rhodamine, 100 mM Try). After 12 hours, fluorescence distribution was visualized and quantified using the IVIS system with excitation/emission wavelengths set at 748 nm and 780 nm, respectively.

**Bulk RNA sequencing.**

To investigate the transcriptional changes induced by the treatment, bulk RNA sequencing was performed on both treated macrophages and untreated control cells. Total RNA was extracted from all samples utilizing the RNeasy Micro Kit (Qiagen). The integrity and concentration of the RNA were evaluated with an Agilent TapeStation 2200 system. Only samples demonstrating high RNA integrity (RIN > 6.0) were advanced to the subsequent library preparation stage. Sequencing libraries were constructed using the Kapa mRNA Hyperprep Kit, optimized for Illumina sequencing systems. The prepared libraries were then pooled, and their concentration was accurately quantified via quantitative PCR. Finally, the pooled libraries were sequenced on an Illumina NextSeq platform operated in high-output mode, generating 38 bp paired-end reads. A detailed description of the bioinformatic analysis pipeline is provided in the following sections:

1. RNA Extraction and Quality Control: Tumor tissues were harvested from mice following a three-day treatment period. Total RNA was isolated from these tissues using TRIzol® Reagent as per the manufacturer's protocol (Invitrogen). Subsequent removal of genomic DNA was performed with DNase I (TaKara). The quality of the purified RNA was assessed on a 2100 Bioanalyzer (Agilent), and concentration was measured using a ND-2000 spectrophotometer (NanoDrop Technologies). Only high-integrity RNA samples, meeting stringent criteria (OD260/280=1.8-2.2, OD260/230≥2.0, RIN≥6.5, 28S:18S ratio≥1.0, and total quantity >1μg), were utilized for library construction.
2. Library Preparation and Sequencing: RNA-seq transcriptome libraries were prepared from 1μg of high-quality total RNA using the TruSeqTM RNA sample preparation Kit (Illumina). The work-flow involved isolation of messenger RNA via polyA selection with oligo(dT) beads, followed by fragmentation. First-strand and second-strand cDNA were synthesized using a SuperScript double-stranded cDNA synthesis kit (Invitrogen) with random hexamer primers. The resulting cDNA fragments underwent end-repair, adenylation, and ligation of Illumina adapters. Following this, libraries were size-selected for fragments of approximately 300 bp on a 2% Low Range Ultra Agarose gel. The selected fragments were then PCR-amplified for 15 cycles using Phusion DNA polymerase (NEB). After quantification, the final libraries were sequenced on an Illumina HiSeq X Ten/NovaSeq 6000 platform to generate paired-end reads of 150 bp in length.
3. Read Processing and Alignment: Raw sequencing reads were first processed to remove adapter sequences and low-quality bases using SeqPrep and Sickle with their default parameters. The resulting high-quality clean reads were then aligned to the reference genome in an orientation-aware manner using the HISAT2 software. The mapped reads for each sample were subsequently assembled into transcripts using StringTie in a reference-guided mode.
4. Differential Expression and Functional Analysis: Gene expression levels were quantified using the TPM (transcripts per million reads) method, with RSEM software employed for accurate abundance estimation. Differential expression analysis between comparison groups was conducted using DESeq2/DEGseq/EdgeR. Genes meeting the thresholds of |log2FoldChange| > 1 and a statistically significant adjusted p-value (Q value ≤ 0.05 for DESeq2/EdgeR or Q value ≤ 0.001 for DEGseq) were classified as significantly differentially expressed genes (DEGs). To interpret the biological functions of these DEGs, Gene Ontology (GO) enrichment and Kyoto Encyclopedia of Genes and Genomes (KEGG) pathway analyses were performed. Functional terms and pathways with a Bonferroni-corrected p-value ≤ 0.05, compared to the whole-transcriptome background, were considered significantly enriched. These analyses were implemented with Goatools and KOBAS, respectively.
5. Alternative Splicing Analysis: Alternative splicing events were systematically identified and quantified from the RNA-seq data using the rMATS software. The analysis focused on detecting five primary types of splicing variations: skipped exons, alternative 5' splice sites, alternative 3' splice sites, mutually exclusive exons, and retained introns. Only splicing events supported by isoforms that were either consistent with known references or contained novel splice junctions were considered for further investigation.

**Western blot analysis**

Cells (2 × 10⁵ per well) were seeded in six-well plates and subjected to the respective treatment conditions for 24 hours. Total proteins were then extracted and quantified in accordance with the manufacturer’s protocol. For Western blot analysis, 40 μg of protein per sample was separated by electrophoresis, transferred to a membrane, and incubated with the appropriate primary and secondary antibodies. Protein bands were visualized using a standard chemiluminescence detection system and quantified based on the recommended procedure.

**In vivo experiments**

All mice were kept in accordance with the policies on animal research of the National Ministry of Health. All mice used for animal experiments are female with a gentle character, and the sex of the mice does not affect the results of the experiments. C57 (6-7 weeks old) were procured from Charles River Laboratories. Animals were maintained under 12 h light and dark cycles with food and water ad libitum.

To establish subcutaneous 4T1 breast tumor models, 5 × 10⁵ 4T1 cells were implanted into the upper surface of the hind leg of female Babl/c mice. Tumor dimensions were measured every two days, and volumes were calculated according to the formula: volume = 0.5 × length × width². For orthotopic pancreatic tumor models, 1.5 × 10⁵ Panc-02-Luc cells were surgically injected into the pancreas of C57 mice. Tumor progression was monitored via the IVIS® imaging system (VISQUE Invivo Smart-LF, Korea).

For therapeutic evaluation, treatment was initiated when subcutaneous tumor volumes reached an average of 50 mm³ or when the average bioluminescence signal of orthotopic pancreatic tumors attained between 5 × 10⁸ and 1 × 10⁹ p/s/cm²/sr. Mice were intravenously administered saline, JQ1@NPs-aCD11b, Try@NPs-aCD11b, JT@NPs-aCD11b or JT@NPs-aCD11b + CL. Animals received two doses administered 7 days apart. Humane endpoint criteria included tumor diameter exceeding 15 mm or the presence of ulceration, at which point mice were euthanized. Tumor progression and survival rates were monitored via the IVIS imaging system. One week post-treatment, animals were humanely sacrificed, and tumor specimens were harvested for subsequent analyses:

(i) **Assessment of tumor apoptosis**: Tumor samples were fixed in 4% paraformaldehyde and embedded in paraffin. Sections of 8 μm thickness were deparaffinized, permeabilized, and subjected to apoptosis detection using the DeadEnd Fluorometric TUNEL assay (Promega), followed by nuclear staining with DAPI. Fluorescent images were acquired using confocal laser scanning microscopy (Olympus FV3000).

(ii) **Immunofluorescence staining**: Paraffin-embedded tumor sections (8 μm) were processed by Wuhan Servicebio Technology Co. Ltd. Following antigen retrieval, permeabilization, and blocking with 5% BSA, tissue sections were incubated with specific antibodies to identify M1 and M2 TAMs via CLSM.

(iii) **Immune cell profiling**: Single-cell suspensions were prepared from dissociated tumor tissues according to established protocols. After erythrocyte lysis with ACK buffer, cells were stained with fluorophore-conjugated antibodies and analyzed by flow cytometry to quantify TAMs, activated dendritic cells, effector T cells, NK cells, myeloid-derived suppressor cells (MDSCs), and regulatory T cells (Tregs).

(iv) **Cytokine and chemokine measurement**: Tumor homogenates were prepared using mechanical disruption. The mRNA expression levels of cytokines and chemokines in the supernatant were assessed by quantitative RT-PCR, while protein concentrations were determined using commercial ELISA kits according to the manufacturer's protocols.

**Isolation of Macrophages via Magnetic-Acted Cell Sorting (MACS)**

Pancreatic tumor specimens were processed into single-cell suspensions according to the manufacturer's guidelines. Pancreatic immune cells, both untreated and following JT@NPs-aCD11b treatment, were harvested. Macrophages were subsequently isolated from the tumor tissue through negative selection, employing the MojoSort™ Mouse Macrophage Isolation Kit (BioLegend, USA) in conjunction with a magnetic separation device. The isolated macrophages were cultured in RPMI 1640 medium supplemented with 10% fetal bovine serum (FBS) for subsequent functional studies, unless specified otherwise.

**Single-Cell RNA Sequencing: Library Preparation and Data Generation**

Macrophage-specific single-cell RNA sequencing (scRNA-seq) libraries were prepared and sequenced on the BD Rhapsody™ platform (Becton, Dickinson and Company, NJ, USA), following the standard protocol provided by OE Biotech Co., Ltd. (Shanghai, China). Raw sequencing data were processed through the BD Rhapsody Analysis Pipeline to generate gene expression matrices. This process involved demultiplexing cellular barcodes, aligning reads to the reference genome and transcriptome with the STAR aligner, and normalizing read counts across all samples.

**Processing and Integration of scRNA-seq Data**

The gene expression data were imported and processed using the Seurat package (v4.4.0). A stringent quality control step was implemented, excluding cells with fewer than 500 or more than 5,000 detected genes, a mitochondrial gene content exceeding 10%, a total UMI count below 500, an erythrocyte content higher than 3%, and genes expressed in fewer than three cells. Potential ambient RNA contamination and doublets were further addressed using the SoupX (v1.6.2) and DoubletFinder (v2.0.3) packages, respectively. The filtered data were log-normalized and scaled, regressing out variations attributable to sequencing depth and mitochondrial percentage. Data integration was performed using 2,000 variable features as anchors and 40 principal dimensions. Cell clusters were identified using the "FindClusters" function at a resolution of 0.1, and the results were visualized in two dimensions using Uniform Manifold Approximation and Projection (UMAP). For subpopulation analysis, cells were subset and re-clustered using the first 30 principal components, with resolution adjusted for optimal separation. Cell types were annotated based on established marker genes. Additionally, scRNA-seq data from a PANC mouse model treated with Mip@MΦ were integrated into the primary dataset using the project LSI method in R.

**Functional Enrichment and Pathway Analysis**

Differentially expressed genes (DEGs) across clusters were identified with the "FindAllMarkers" function in Seurat. Gene Set Enrichment Analysis (GSEA) was conducted using the fGSEA package (v1.24.0). Gene Ontology (GO) term enrichment analysis was performed with the clusterProfiler and data.table R packages. The "AddModuleScore" function in Seurat was used to calculate signature scores for gene sets obtained from the MSigDB database.

**Trajectory Inference Using Monocle3**

Developmental trajectories of macrophages were reconstructed with Monocle3 (v1.0.0). The gene expression matrix was imported from Seurat to create a CellDataSet object. Variable genes significantly associated with the trajectory (q-value < 0.01) were selected as ordering genes using the "differentialGeneTest" function. Dimensionality reduction was performed with the "reduceDimension" function (method = 'DDRTree'), without prior normalization, to project the cells into a pseudotime trajectory. A heatmap was generated to visualize key genes dynamically expressed along the inferred progression.

**Analysis of Differential Gene Expression**

The "FindMarkers" function in Seurat (parameters: logfc.threshold = 0, min.pct = 0.35) was applied to identify genes that were differentially expressed between normal and pathological conditions. The results were visualized using radar and scatter plots to illustrate the number of DEGs within each cellular subpopulation.

**Assessment of Cell-Cell Communication**

Cell-cell interaction networks among macrophage subtypes were interrogated using the CellChat package (v1.6.1) with its default parameters, to infer potential intercellular signaling pathways.

**Data availability statement.** The authors declare that all data needed to support the findings of this study are provided within the article, Supplementary information, and Source data file. This study utilizes publicly accessible data from the Protein Data Bank (PDB) under accession code: 1DGF (https://doi.org/10.2210/pdb1DGF/pdb). A reporting summary for this article can be found in the Supplementary Information file. The raw data of RNA-sequencing has been deposited in the China National Center for Bioinformation under the BioProject accession number PRJCA051069 (<https://www.cncb.ac.cn/>).

***Supporting Figures***

**Figure S1.** Expression of proinflammatory cytokines (a CCL22, b IL‑10, c TNF‑α, and d IL‑1β) in bone marrow‑derived macrophages (BMDMs) measured by enzyme‑linked immunosorbent assay (ELISA) after different treatments. Data were expressed as the mean ± SD. (n = 5; ****P < 0.0001).

**Figure S2.** Analysis of differentially expressed genes in tumor cells after Trypsinogen treatment using (a) Gene Ontology (GO) classification and (b) Gene and Genome Encyclopedia (KEGG) enrichment technology.

**Figure S3.** Quantitative statistical results of mean immunofluorescence intensity for different markers within cells after Try treatment. Data were expressed as the mean ± SD. (n = 3; ****P < 0.0001).

**Figure S4.** Qualification of protein bands in Figure 3j. Data were expressed as the mean ± SD. (n = 3; ns, no significant, ****P < 0.0001).

**Figure S5.** The expression of proinflammatory cytokines in BMDMs was measured using enzyme-linked immunosorbent assay (ELISA). Prior to Try stimulation, the cells were pretreated with the following pharmacological inhibitors: (a, b) CA-074 Me (a selective cathepsin B inhibitor, 50 μM) to block Try activation; (c, d) MCC950 (a potent NLRP3 inflammasome inhibitor, 50 μM) to inhibit NLRP3 inflammasome assembly and activation; (e, f) IL-1 receptor antagonist (IL-1Ra, 25 nM) to block autocrine IL-1β signaling; and (g, h) BAY 11-7082 (an NF-κB pathway inhibitor, 10 μM) to suppress NF-κB activation. Data were expressed as the mean ± SD. (n = 5; ****P < 0.0001).

**Figure S6.** The change in size of JT@Lips incubated in PBS containing 10% fetal bovine serum (FBS) at 37°C over 70 h. Data were expressed as the mean ± SD. (n = 3).

**Figure S7.** Quantitative statistics of the proportion of FITC-positive cells analyzed by flow cytometry. Data were expressed as the mean ± SD. (n = 3; ***P < 0.001, ****P < 0.0001).

**Figure S8.** Quantitative statistical results of mean immunofluorescence intensity for FITC within cells after coincubated with FITC-labeled JT@NPs-aCD11b for different times. Data were expressed as the mean ± SD. (n = 3; ****P < 0.0001).

**Figure S9.** Quantitative statistical results of mean immunofluorescence intensity for different markers within cells after different treatments. Data were expressed as the mean ± SD. (n = 3; no significant, ****P < 0.0001).

**Figure S10.** (a) Gating strategies for the (b) M2 (F4/80^+^ CD206^+^) and (c) M1 (F4/80^+^ CD86^+^) BMMs after different treatments.

**Figure S11.** Quantitative analysis of phagocytosis rates in Panc02 cancer cells determined by flow cytometry. Data were expressed as the mean ± SD. (n = 3; no significant, ****P < 0.0001).

**Figure S12.** The expression of CD47 on Panc02 cells after various treatments was measured by flow cytometry.

**Figure S13.** The time‑dependent changes in proinflammatory cytokines (a) TNF‑α and (b) IL‑6 levels in mice after different treatments were determined by ELISA. Data were expressed as the mean ± SD. (ns, no significant).

**Figure S14.** Biodistribution of nanoformulations in major organs and tumors at 12 hours after intravenous injected in Panc-2 tumor-bearing mice. Data were expressed as the mean ± SD. (n = 3; **P < 0.01, ****P < 0.0001).

**Figure S15.** Survival in the PANC mice model after different treatments (n = 10).

**Figure S16.** Immunofluorescence staining for (a) F4/80/Arg1 positive and F4/80/iNOS positive cells in tumor tissuess from different groups and corresponding (b, c) quantative analysis results. Data were expressed as the mean ± SD. (n = 3; ns, nosignificant, *P < 0.05, **P < 0.01, ****P < 0.0001).

**Figure S17.** Representative flow dot plots of TEM and TCM in the splenic lymphocytes analyzed by FC.

**Figure S18.** The contents of various cytokines in tumor tissues of mice after different treatments measured by ELISA. Data were expressed as the mean ± SD. (n = 3; ns, nosignificant, ****P < 0.0001).

**Figure S19.** Immunohistochemical detection of CRT, HSP70, and HMGB1 in tumor tissues across different groups.

**Figure S20.** Immunohistochemical staining images of the TUNEL nick-end labeling assay of tumor tissue from different groups and corresponding quantative analysis results. (n = 3; **P < 0.01, ****P < 0.0001).

**Figure S21.** Survival in the PANC mice model after different treatments (n = 10).

**Figure S22.** Immunofluorescence staining for (a) F4/80/Arg1 positive and F4/80/iNOS positive cells in tumor tissuess from different groups and corresponding (b) quantative analysis results. Data were expressed as the mean ± SD. (n = 3; ns, nosignificant, *P < 0.05, **P < 0.01, ****P < 0.0001).

**Figure S23.** The quantification of immunosuppressive cells such as (a) MDSCs and (b) Tregs and immunostimulatory cells such as (c) NK cells, (d) activated dendritic cells (DCs), (e, f) CD8+ cytotoxic and memory T cells, and (g, h) CD4+ helper and memory T cells in tumors derived from different groups analyzed by flow cytometry. Data were expressed as the mean ± SD. (n = 3; ns, nosignificant, *P < 0.05, **P < 0.01, ****P < 0.0001).

**Figure S24.** Quantitative statistical analysis for the ratio of apoptositc cell of tumor cells in different groups. Data were expressed as the mean ± SD. (n = 3; ns, nosignificant, ***P < 0.001, ****P < 0.0001).
